# Synthesis and Cytotoxic Activity of the Derivatives of *N*-(Purin-6-yl)aminopolymethylene Carboxylic Acids and Related Compounds

**DOI:** 10.3390/molecules28041853

**Published:** 2023-02-15

**Authors:** Victor P. Krasnov, Olga A. Vozdvizhenskaya, Maria A. Baryshnikova, Alexandra G. Pershina, Vera V. Musiyak, Tatyana V. Matveeva, Kseniya V. Nevskaya, Olga Y. Brikunova, Dmitry A. Gruzdev, Galina L. Levit

**Affiliations:** 1 Postovsky Institute of Organic Synthesis, Russian Academy of Sciences (Ural Branch), 620108 Ekaterinburg, Russia; 2 Blokhin National Medical Research Center of Oncology, Ministry of Health of the Russian Federation, 115522 Moscow, Russia; 3 Center of Bioscience and Bioengineering, Siberian State Medical University, 634050 Tomsk, Russia; 4 Research School of Chemical and Biomedical Engineering, National Research Tomsk Polytechnic University, 634050 Tomsk, Russia

**Keywords:** purine, omega-amino acids, 7,8-difluoro-3,4-dihydro-3-methyl-2*H*-[1,4]benzoxazine, cytotoxicity, tumor cells

## Abstract

Testing a number of *N*-[omega-(purin-6-yl)aminoalkanoyl] derivatives of 7,8-difluoro-3,4-dihydro-3-methyl-2*H*-[1,4]benzoxazine in a panel of nine tumor cell lines has shown that the studied compounds exhibit high cytotoxic activity, especially against 4T1 murine mammary carcinoma, COLO201 human colorectal adenocarcinoma, SNU-1 human gastric carcinoma, and HepG2 human hepatocellular carcinoma cells. Synthesis and study of structural analogs of these compounds made it possible to find that the presence of both a difluorobenzoxazine fragment and a purine residue bound via a linker of a certain length is crucial for the manifestation of the cytotoxic activity of this group of compounds. The study of the effect of the most promising compound on the cell cycle of the human tumor cell lines, the most sensitive and least sensitive to cytotoxic action (MDA-MB-231 breast adenocarcinoma and COLO201 colorectal adenocarcinoma, respectively), allows us to conclude that this compound is an inhibitor of DNA biosynthesis. The found group of purine conjugates may be of interest in the design of new antitumor agents.

## 1. Introduction

The prevalence of cancer in the world is enormous. Despite advances in the field of tumor therapy, the number of reported cases of cancer is increasing every year. According to the International Agency for Research on Cancer, approximately 19.3 million new cases of cancer have been identified in 2020 [1]. The design of new agents for cancer treatment, primarily of the most lethal forms, is an urgent task. Preparation of new compounds that are highly toxic to tumor cells and are well tolerated at therapeutic doses can lay the foundation for new cancer treatment regimens and contribute to the reduction in cancer mortality.

**Figure 1:**
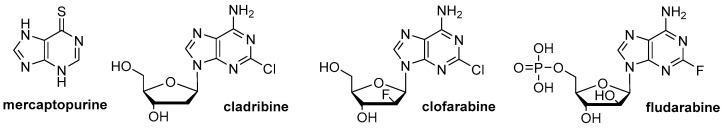
Purine-based anticancer agents.

Purine-containing compounds, such as nucleic acids, ATP, ADP, and NAD, play a very important role in cell metabolism and vital activity. Many compounds with antineoplastic activity have been found among purine derivatives. Purine-based compounds such as mercaptopurine, cladribine, clofarabine, and fludarabine (Figure 1) are among the clinically used anticancer agents.

**Figure 2:**
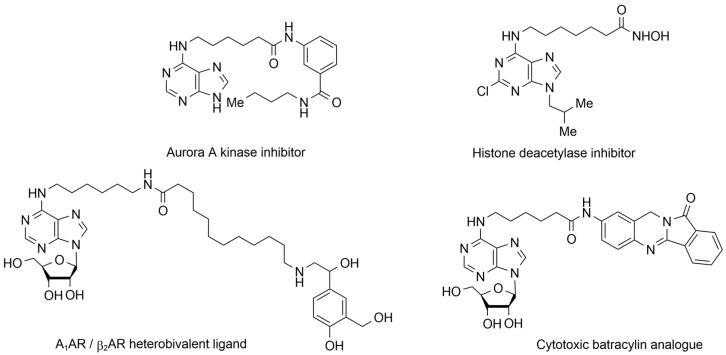
Selected literature examples of biologically active purine conjugates containing fragments of polymethylene carboxylic acids. Aurora A kinase inhibitor [15], histone deacetylase inhibitor [18], A_1_AR/β_2_AR heterobivalent ligand [20], cytotoxic batracylin analogue [21].

In recent decades, efficient cytostatics with different mechanisms of action have been found among purine derivatives: inhibitors of cyclin-dependent kinases [2,3,4,5], apoptosis inducers [6,7,8,9,10] and some others [11,12,13,14]. Purine conjugates containing fragments of polymethylene carboxylic acids deserve special attention. It has been reported that these compounds are kinase inhibitors [15,16,17], histone deacetylase inhibitors with antiproliferative activity [18], and also bivalent modulators of A_1_ adenosine receptors (A_1_ARs) and β_2_-adrenergic receptors (β_2_ARs) [19,20] (Figure 2). At the same time, it has been reported that the introduction of an adenosine residue leads to a significant decrease in the toxicity of the ω-aminopolymethylenecarbonyl derivatives of batracylin [21].

**Figure 3:**
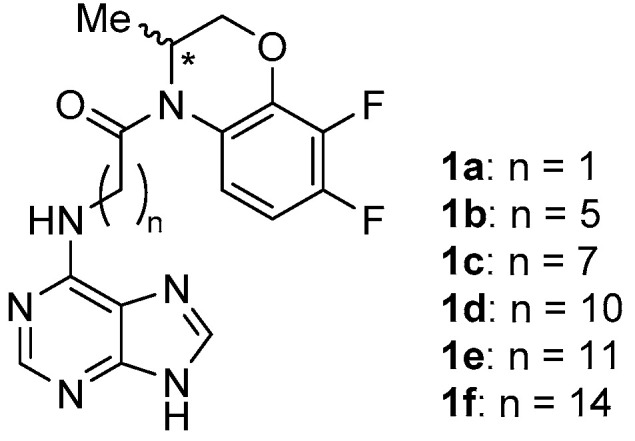
Structures of the previously obtained purine conjugates with 7,8-difluoro-3,4-dihydro-3-methyl-2*H*-[1,4]benzoxazine.

In recent years, we have focused on the preparation of new purine and 2-aminopurine derivatives and the search for highly bioactive compounds in this series [22,23,24,25,26,27,28,29,30]. In particular, we have synthesized a number of purine conjugates with various *N*-heterocycles attached via a linker, the omega-amino acid residue, –NH(CH_2_)_n_CO–, to position 6 of the purine nucleus [22,23,24,25,26,27,28,29]. The results of biological testing have shown that conjugates of purine with racemic 7,8-difluoro-3,4-dihydro-3-methyl-2*H*-[1,4]benzoxazine (Figure 3, compounds **1a–f**) attached via 6-aminohexanoyl (*n* = 5, compound **1b**) or 8-aminooctanoyl (*n* = 7, compound **1c**) residues, as well as (*S*)-enantiomer of compound **1b**, exhibit high antiviral activity in vitro against herpes simplex virus type 1 (HSV-1), including acyclovir-resistant HSV-1 strain [26,27,28].

It has also been found that some of the synthesized compounds exhibit high cytotoxicity. Thus, the concentration of compound **1d** (*n* = 10), which caused the death of 50% Vero E6 cells, was less than 30 µM [28]. In the framework of this work, in order to elucidate the structure–activity relationship, we have synthesized and studied the cytotoxic activity of purine conjugates containing fragments of omega-amino acids with different lengths of the polymethylene chain as linkers, and related compounds against tumor cell lines.

## 2. Results and Discussion

### 2.1. Chemistry

**Scheme 1:**
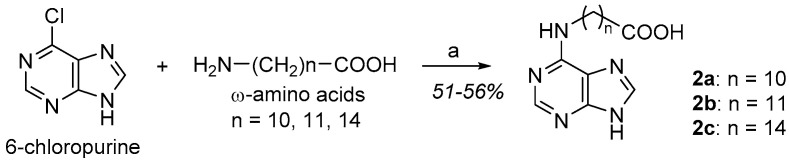
Synthesis of *N*-(purin-6-yl)amino carboxylic acids **2a–c**. (a) Na_2_CO_3_, H_2_O, Δ, 3 h.

Synthesis of the studied compounds **1a–c**, (*S*)-**1b** [26], and **1d–f** [28] was described earlier. To reveal the structure–activity relationship, we synthesized a number of related compounds. Thus, *N*-(purin-6-yl)amino acids **2a–c** were synthesized in moderate yields by analogy with a literature method [31], reacting the corresponding omega-amino acids with 6-chloropurine in an aqueous Na_2_CO_3_ solution under reflux (Scheme 1).

**Scheme 2:**
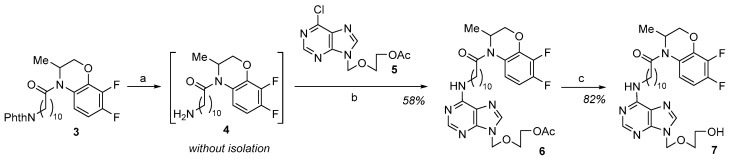
Synthesis of *N*(9)-substituted purine conjugates **6** and **7**. (a) NH_2_NH_2_×H_2_O, EtOH, Δ, 2 h; (b) NEt_3_, *n-*BuOH, Δ, 6 h; (c) 1 N NaOH, EtOH, rt, 1 day.

A major disadvantage of compound **1d**, which limits its further study, is its low solubility in aqueous media. In order to increase the solubility and, possibly, to improve the profile of the cytotoxic action, we synthesized compound **7** containing a 2-hydroxyethoxymethyl fragment in position *N*(9) of purine (Scheme 2). Compounds **6** and **7** were synthesized starting from *N*-phthaloyl derivative **3** [28]. At the first step, compound **3** was treated with hydrazine hydrate in refluxing EtOH to afford to the corresponding amine **4**, which (without isolation) was introduced into the reaction of nucleophilic substitution of chlorine in 9-[(2-acetoxyethoxy)methyl]-6-chloropurine (**5**) [32], thus resulting in compound **6**. The subsequent alkaline hydrolysis in 1N NaOH gave the target compound **7** in good yield.

**Scheme 3:**
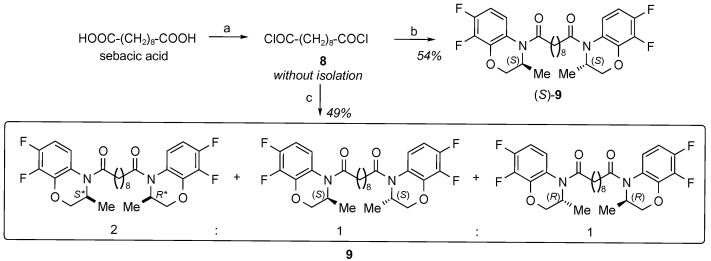
Synthesis of sebacates **9**. (a) SOCl_2_, Δ, 1 h; (b) (*S*)-7,8-difluoro-3,4-dihydro-3-methyl-2*H*-[1,4]benzoxazine, PhNEt_2_, CH_2_Cl_2_, rt, 2 days; (c) (*RS*)-7,8-difluoro-3,4-dihydro-3-methyl-2*H*-[1,4]benzoxazine, PhNEt_2_, CH_2_Cl_2_, rt, 2 days.

For comparative studies, we have obtained two compounds without purine residue, but the polymethylenecarboxylic acid amide fragment is retained (Scheme 3). As a result of the interaction of sebacoyl dichloride (**8**) with enantiomerically pure (*S*)-7,8-difluoro-3,4-dihydro-3-methyl-2*H*-[1,4]benzoxazine [33] in dichloromethane at room temperature in the presence of *N*,*N*-diethylaniline (PhNEt_2_) as a HCl acceptor, diamide (*S*)-**9** was obtained; the reaction with racemic 7,8-difluoro-3,4-dihydro-3-methyl-2*H*-[1,4]benzoxazine gave a mixture of three diastereomers **9**. Using chiral HPLC, the (R*,S*)–(*S*,*S*)–(*R*,*R*) diastereomeric ratio has been found to be approximately 2:1:1 (see the Supplementary Materials, Figures S19 and S20).

### 2.2. Cytotoxicity Assessment

The cytotoxicity of the test compounds was studied using the MTT assay [34] against a number of cell lines: CT-26 murine colon carcinoma, 4T1 murine mammary carcinoma, MDA-MB-231 human breast adenocarcinoma, COLO201 human colorectal adenocarcinoma, HepG2 human hepatocellular carcinoma, A549 human non-small-cell lung carcinoma, SK-BR-3 human breast adenocarcinoma, SNU-1 human gastric carcinoma, Jurkat human acute T-lymphoblastic leukemia, and WI-38 human lung fibroblasts (normal cells). Doxorubicin (Dox) was used as a reference drug. Cells were incubated with test compounds for 72 h.

Since primary data on cytotoxicity were obtained for compounds **1c–f** (Figure 3) [28], we first carried out a detailed study of the cytotoxic activity of these compounds against various cells and determined their 50% cytotoxic concentration (CC_50_) and selective cytotoxicity index (SCI) (Table 1). Selective cytotoxicity index (SCI) (see, for example [35,36]) generally refers to the ability of a test compound to preferentially kill cancer cells, causing less or insignificant damage to normal cells (in our case, WI-38 human lung fibroblasts).

The COLO201 human colorectal adenocarcinoma and 4T1 murine mammary carcinoma cell lines were found to be more sensitive to compounds **1c–f** (in some cases, CC_50_ < 1 µM), while the cell lines of SK-BR-3 and MDA-MB-231 human breast adenocarcinomas, as well as A549 human non-small-cell lung carcinoma, were the most resistant to the cytotoxic effects of the studied compounds (CC_50_ > 10 µM).

It should be emphasized that normal cells (WI-38 fibroblasts) are less sensitive to the highest of the studied concentrations (1 × 10^–4^ M) of compounds **1c–f** than tumor cells (Table 2), which allows us to talk about the selectivity of action of these compounds. Compound **1d** exhibits a high cytotoxic activity against a larger number of studied cell lines compared to compounds **1c,e,f**, which makes it a promising candidate for further studies of antitumor activity.

The cytotoxicity of compounds **2a–c**, **6**, **7**, and **9** was also studied using the MTT assay against a number of tumor cell lines: A549, SK-BR-3, SNU-1, Jurkat, as well as normal fibroblasts WI-38 (Table 3).

Compound **1a**, in which purine and benzoxazine residues are separated by a single methylene group, was found to be non-cytotoxic (Table 3). Compound **1b**, the linker of which contains five methylene groups, showed cytotoxic activity, but in general, it was inferior to compound **1d**. It turned out that SK-BR-3 breast cancer cells were resistant to compounds **1b** and (*S*)-**1b** (CC_50_ > 1 × 10^–4^), in contrast to compound **1d**. The cytotoxic activity of the enantiomer (*S*)-**1b** practically did not differ from that of racemate **1b** (except for the A549 cell line).

Among purine conjugates with omega-amino acids, compounds **2a,b** were cytotoxic only against the Jurkat cell line (Table 3). Compound **2c** showed no cytotoxic activity. Sebacates (*S*)-**9** and **9** that do not contain a purine fragment, did not exhibit cytotoxic activity.

Apparently, the presence of both a purine fragment and difluorobenzoxazine connected via a linker of a certain length is important for the manifestation of cytotoxic activity.

Compounds **6** and **7** containing 2-acetoxy- and 2-hydroxy-ethoxymethyl fragments, respectively, exhibited approximately the same cytotoxicity as the most active compound **1d**. It was not possible to achieve a significant increase in the solubility of conjugate **7** compared to compound **1d**. At the same time, the preservation of the activity of compound **7** compared to **1d** indicates that the modification at position 9 of purine does not lead to a loss of cytotoxic activity, so further introduction of hydrophilic groups, for example, sugar residues, can result in water-soluble conjugates.

### 2.3. Cell Cycle Analysis

To study the potential cytostatic effect of compound **1d**, we chose the method of cell cycle analysis using flow cytometry by DNA staining with propidium iodide [37].

**Figure 4:**
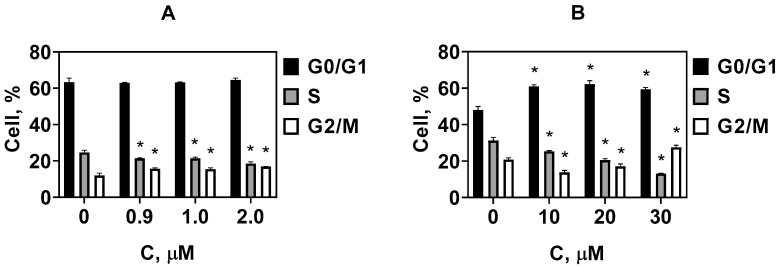
Effect of compound **1d** on the cell cycle of the (**A**) COLO201 and (**B**) MDA-MB231 cells. Data are presented as the mean ± SD (*n* = 3). * *p* ˂ 0.05 compared to control.

Based on experiments to determine the cytotoxic properties of compound **1d**, the following two cell lines were selected for study: MDA-MB-231, which showed high resistance to the action of compound **1d**, and COLO201, which showed the highest sensitivity among all tested lines. To analyze possible effects on the cell cycle, we tested three concentrations of compound **1d**, which were close to CC_50_ calculated on the basis of viability assessment data (MTT assay). Figure 4 shows the phase distribution of the cell cycle of COLO201 and MDA-MB231 cells after incubation with compound **1d** for 24 h.

According to the data obtained, compound **1d** at concentrations close to CC_50_ after incubation for 24 h, had a dose-dependent cytostatic effect on COLO201 and MDA-MB-231 cell lines: a decrease in the proportion of cells in the S phase was observed (Figure 4A,B). In the case of COLO201, cell cycle arrest occurred in the G2/M phase; however, the cell proliferation index was in the range 35–37% and not changed compared to the control (see the Supplementary Materials, Table S2). For the MDA-MB-231 cell line, a decrease in the proliferation index (see the Supplementary Materials, Table S3) and arrest of the cell cycle in the G0/G1 phase were observed over the entire range of concentrations studied; when the concentration of **1d** was increased to 30 µM, an increase in the proportion of cells in the G2/M phase was observed (Figure 4B).

When analyzing the possible mechanism of antitumor activity, it can be concluded that compound **1d** at a dose lower CC_50_ blocks DNA synthesis in cells. This leads to the accumulation of cells in the G1 phase. An increase in the concentration of the substance above CC_50_ causes a pronounced death of cells in the G1 phase, which manifests itself in an increase in the relative proportion of cells in the G2/M phase with a progressive reduction in cells in the S phase. The delay in the G2/M phase is most likely due to the lengthening of the DNA repair process. It is important to note that the delay of cells in the G2/M phase leads to an increase in the calculated value of the cell proliferation index; thus, despite a pronounced cytostatic effect, no change in this indicator was recorded for COLO201 cells. Thus, compound **1d** can be considered as a blocker of DNA synthesis.

## 3. Materials and Methods

### 3.1. Chemistry

#### 3.1.1. Chemistry General Section

Compounds **1a–c**, (*S*)-**1b** [26], **1d–f**, **3** [28], and **5** [32] were obtained as previously described. Other reagents are commercially available and were purchased from Alfa Aesar (Heysham, UK). Melting points were obtained on a SMP3 apparatus (Barloworld Scientific, Staffordshire, UK) and are uncorrected. Optical rotations were measured on a Perkin Elmer M341 polarimeter (Perkin Elmer, Waltham, MA, USA). The reactions were monitored by thin layer chromatography (TLC) using silica gel precoated Sorbfil plates (Imid, Krasnodar, Russia); compounds were visualized by UV irradiation at 254 nm and iodine vapors. Flash column chromatography was performed using Silica gel 60 (230–450 mesh) (Alfa Aesar, Heysham, UK). The ^1^H, ^19^F, and ^13^C NMR spectra were recorded on a Bruker AVANCE 500 spectrometer (Bruker, Karlsruhe, Germany). Chemical shifts are given in ppm and are referenced to TMS (or DSS) and hexafluorobenzene as internal standards and multiplicities are reported as s (singlet), d (doublet), t (triplet), and m (multiplet). The ^1^H and ^19^F NMR spectra of compounds **6** and **7** were recorded in DMSO-*d*_6_ at 100 °C; the ^1^H and ^13^C NMR spectra of compounds **2b,c** were recorded in a D_2_O–NaOD mixture at ambient temperature. For NMR spectra of the compounds obtained, see the Supplementary Materials, Figures S1–S18. CHN-Elemental analysis was performed using Perkin Elmer 2400 II analyzer (Perkin Elmer, Waltham, MA, USA). High resolution mass spectra were obtained on a Bruker maXis Impact HD mass spectrometer (Bruker, Karlsruhe, Germany), electrospray ionization (ESI) with direct sample inlet (4 L/min flow rate). Analytical chiral HPLC of compounds (*S*)-**9** and **9** was performed on an Agilent 1100 instrument (Agilent Technologies, Santa Clara, CA, USA) using a (S,S)-Whelk-O1 column (250 × 4.6 mm, 5 µm) (Phenomenex, Torrance, CA, USA); flow rate 0.8 mL/min, detection at 280 nm. For HPLC data for compounds (*S*)-**9** and **9**, see the Supplementary Materials, Figures S19 and S20.

#### 3.1.2. Synthesis

##### General Procedure for the Synthesis of N-(Purin-6-yl)amino Carboxylic Acids **2a–c**

6-Chloropurine (2.00 g, 12.9 mmol) and Na_2_CO_3_ (1.37 g, 12.9 mmol) were added to a suspension of appropriate ω-amino acid (25.9 mmol) in water (40 mL). The reaction mixture was refluxed for 3 h; then 4 N HCl (2 mL) was added, the precipitate formed was filtered off and recrystallized from ethanol (in the case of compounds **2a** and **2b**) or washed with hot ethanol (in the case of compound **2c**).

*N-(Purin-6-yl)-11-aminoundecanoic Acid (**2a**)*. Yield 2.10 g (51%). Colorless powder m.p. 172–174 °C (EtOH). ^1^H NMR (500 MHz, DMSO-*d*_6_) δ (ppm): 1.24 (br. s, 8H, 4×CH_2_), 1.28–1.37 (m, 4H, 2×CH_2_), 1.43–1.52 (m, 2H, CH_2_), 1.59–1.64 (m, 2H, CH_2_), 2.18 (dd, *J* = 7.4, 7.4 Hz, 2H, CH_2_CO), 3.53 (br. s, 2H, CH_2_NH), 8.33 (s, 1H, H-8 purine), 8.41 (br. s, 1H, H-2 purine), 8.82 (br. s, 1H, NH), 12,00 (br. s, 1H, NH purine), 13.65 (br. s, 1H, COOH). ^13^C NMR (125 MHz, DMSO-*d*_6_) δ (ppm): 24.4, 26.2, 28.5, 28.6 (3C), 28.7, 28.8, 33.6, 40.5 (br. s), 114.5 (br. s), 141.3, 148.5, 148.8, 152.1, 174.4. HRMS (ESI): *m*/*z* [M+H]^+^ calcd for [C_16_H_26_N_5_O_2_]^+^: 320.2082, found: 320.2080.

*N-(Purin-6-yl)-12-aminododecanoic Acid (**2b**)*. Yield 2.36 g (55%). Colorless powder m.p. 190–191 °C (EtOH). ^1^H NMR (500 MHz, D_2_O + NaOD) δ (ppm): 1.03 (br. s, 6H, 3×CH_2_), 1.07–1.17 (m, 6H, 3×CH_2_), 1.19–1.25 (m, 2H, CH_2_), 1.45–1.51 (m, 2H, CH_2_), 1.51–1.57 (m, 2H, CH_2_), 2.16 (dd, *J* = 7.7, 7.6 Hz, 2H, CH_2_CO), 3.49 (dd, *J* = 6.5, 6.4 Hz, 2H, CH_2_NH), 7.93 (s, 1H, H-8 purine), 8.15 (s, 1H, H-2 purine). ^13^C NMR (125 MHz, D_2_O + NaOD) δ (ppm): 28.6, 28.7, 31.2, 31.26, 31.31, 31.33 (2C), 31.5, 31.6, 40.4, 43.5, 122.7, 152.3, 154.2, 156.7, 160.7, 186.7. HRMS (ESI): *m*/*z* [M+H]^+^ calcd for [C_17_H_28_N_5_O_2_]^+^: 334.2239, found: 334.2244.

*N-(Purin-6-yl)-15-aminopentadecanoic Acid (**2c**)*. Yield 2.72 g (56%). Colorless powder m.p. 198–199 °C. ^1^H NMR (500 MHz, D_2_O + NaOD) δ (ppm): 1.10 (br. s, 12H, 6×CH_2_), 1.14 (br. s, 6H, 3×CH_2_), 1.22–1.29 (m, 2H, CH_2_), 1.45–1.51 (m, 2H, CH_2_), 1.52–1.58 (m, 2H, CH_2_), 2.14 (dd, *J* = 7.9, 7.7 Hz, 2H, CH_2_CO), 3.47 (dd, *J* = 6.5, 6.5 Hz, 2H, CH_2_NH), 7.89 (s, 1H, H-8 purine), 8.13 (s, 1H, H-2 purine). ^13^C NMR (125 MHz, D_2_O + NaOD) δ (ppm): 28.7, 29.0, 31.6, 31.7, 31.8, 31.9 (6C), 40.3, 43.4, 122.6, 152.0, 152.1, 153.7, 156.4, 160.5, 186.1. HRMS (ESI): *m*/*z* [M+H]^+^ calcd for [C_20_H_34_N_5_O_2_]^+^: 376.2708, found: 376,2711.

*{11-[9-(2-Acetoxyethoxy)methylpurin-6-yl]aminoundecanoyl]-7,8-difluoro-3,4-dihydro-3-methyl-2H-*[1,4]*benzoxazine (**6**)*. Hydrazine monohydrate (0.22 mL, 4.52 mmol) was added to a solution of compound **3** (1.25 g, 2.51 mmol) in EtOH (25 mL). The reaction mixture was refluxed for 2 h, and then evaporated to dryness. The residue was treated with Et_2_O (15 mL) and kept at −16 °C overnight; the resulting precipitate was filtered off; filtrate was evaporated to dryness. The residue was re-dissolved in *n-*BuOH (10 mL); the formed solution was added to a solution of compound **5** (0.27 g, 1.00 mmol) and NEt_3_ (0.42 mL, 3.00 mmol) in *n-*BuOH (10 mL). The reaction mixture was refluxed for 6 h, cooled to room temperature, successively washed with 1N HCl (3 × 10 mL), a saturated aqueous NaCl solution (3 × 15 mL), and water (10 mL), and evaporated to dryness under reduced pressure. The residue was purified by flash column chromatography on silica gel (chloroform–EtOH, 98:2 as eluent) to afford 0.35 g (58%) of compound **6** as a colorless oil. ^1^H NMR (500 MHz, DMSO-*d*_6_, 100 °C) δ (ppm): 1.12 (d, *J* = 6.8 Hz, 3H, Me), 1.26 (br. s, 8H, 4×CH_2_), 1.29–1.36 (m, 4H, 2×CH_2_), 1.55–1.65 (m, 4H, 2×CH_2_), 1.91 (s, 3H, CH_3_CO), 2.43–2.49 (m, 1H, CH, partially overlapped with DMSO signal), 2.54–2.60 (m, 1H, CH), 3.56–3.60 (m, 2H, CH_2_), 3.74 (dd, *J* = 5.0, 4.7 Hz, 2H, OCH_2_), 4.08 (dd, *J* = 6.0, 4.7 Hz, 2H, CH_2_OAc), 4.13 (dd, *J =* 11.0, 2.9 Hz, 1H, H-2A benzoxazine), 4.33 (dd, *J =* 11.0, 1.5 Hz, 1H, H-2B benzoxazine), 4.71–4.76 (m, 1H, H-3 benzoxazine), 5.56 (s, 2H, N–CH_2_–O), 6.85 (td, *J =* 9.8, 8.3 Hz, 1H, H-6 benzoxazine), 7.20 (m, 1H, NH), 7.55 (ddd, *J =* 8.3, 5.6, 2.6 Hz, 1H, H-5 benzoxazine), 8.14 (br. s, 1H, H-8 purine), 8.19 (s, 1H, H-2 purine). ^19^F NMR (470 MHz, DMSO-*d*_6_, 100 °C) δ (ppm): 1.98 (ddd, *J* = 20.5, 8.0, 2.0 Hz, 1F, F-8), 20.04–20.14 (m, 1F, F-7). ^13^C NMR (125 MHz, DMSO-*d*_6_) δ (ppm): 15.1, 20.4 (2C), 24.6, 26.3, 28.5, 28.71, 28.73, 28.8, 28.9, 29.0 (br. s), 33.4, 44.8 (br. s), 62.7, 66.8, 69.8, 71.9, 106.7 (d, *J =* 18.0 Hz), 118.8, 119.3, 121.8, 135.7 (d, *J =* 8.5 Hz), 138.9 (dd, *J =* 243.0, 15.5 Hz), 140.7, 146.4 (d, *J =* 244.0 Hz), 148.8 (br. s), 152.9, 154.5, 170.1, 171.0. Calcd (%) for C_30_H_40_F_2_N_6_O_5_: C 59.79, H 6.69, F 6.30, N 13.94. Found (%): C 60.02, H 7.00, F 6.55, N 13.64.

*4-{11-[9-(2-Hydroxyethoxy)methylpurin-6-yl]aminoundecanoyl}-7,8-difluoro-3,4-dihydro-3-methyl-2H-*[1,4]*benzoxazine (**7**)*. The 1N NaOH (1.8 mL) was added to a solution of compound **6** (0.21 g, 0.35 mmol) in EtOH (4 mL). The reaction mixture was stirred at room temperature for 1 day, neutralized with 4 N HCl to pH = 6, and evaporated to dryness. The residue was purified by flash chromatography on silica gel (eluent CHCl_3_–EtOH, 95:5). Yield 0.16 g (82%), colorless oil. ^1^H NMR (500 MHz, DMSO-*d*_6_, 100 °C) δ (ppm): 1.12 (d, *J* = 6.8 Hz, 3H, Me), 1.26 (br. s, 8H, 4×CH_2_), 1.29–1.36 (m, 4H, 2×CH_2_), 1.55–1.65 (m, 4H, 2×CH_2_), 2.43–2.49 (m, 1H, CH, partially overlapped with DMSO signal), 2.54–2.60 (m, 1H, CH), 3.48–3.50 (m, 2H, CH_2_), 3.55–3.60 (m, 4H, 2×CH_2_), 4.13 (dd, *J =* 10.9, 2.9 Hz, 1H, H-2A benzoxazine), 4.25 (br. s, 1H, OH), 4.33 (dd, *J =* 10.9, 1.4 Hz, 1H, H-2B benzoxazine), 4.71–4.76 (m, 1H, H-3 benzoxazine), 5.55 (s, 2H, N–CH_2_–O), 6.84 (td, *J =* 9.9, 8.2 Hz, 1H, H-6 benzoxazine), 7.17 (br. t, *J =* 5.6 Hz, 1H, N**H**CH_2_), 7.55 (ddd, *J =* 7.9, 5.4, 2.4 Hz, 1H, H-5 benzoxazine), 8.13 (br. s, 1H, H-8 purine), 8.19 (s, 1H, H-2 purine). ^19^F NMR (470 MHz, DMSO-*d*_6_, 100 °C) δ (ppm): 1.98 (ddd, *J* = 21.0, 8.0, 2.0 Hz, 1F, F-8), 20.06–20.13 (m, 1F, F-7). ^13^C NMR (125 MHz, DMSO-*d*_6_) δ (ppm): 15.1, 24.6, 26.3, 28.5, 28.71, 28.73, 28.8, 28.9, 29.0 (br. s), 45.0 (br. s), 59.8 (2C), 69.8, 70.7 (2C), 72.1, 106.8 (d, *J =* 18.0 Hz), 118.8 (br. s), 119.3, 121.8, 135.7 (dd, *J =* 10.0, 2.5 Hz), 138.9 (dd, *J =* 243.5, 15.5 Hz), 140.7, 146.5 (d, *J =* 243.5 Hz), 148.9 (br. s), 152.8, 154.5, 171.0. HRMS (ESI): *m*/*z* [M+H]^+^ calcd for [C_28_H_39_F_2_N_6_O_4_]^+^: 561.2995, found: 561.2999.

*General Procedure for the Synthesis of Sebacates **9**.* Thionyl chloride (3.6 mL, 49.62 mmol) was added to sebacic acid (2.00 g, 9.89 mmol); the reaction mixture was refluxed for 1 h, then evaporated to dryness. The residue was dissolved in CH_2_Cl_2_ (25 mL); *N*,*N*-diethylaniline (3.2 mL, 20.0 mmol) and (*S*)-enantiomer or racemic 7,8-difluoro-3,4-dihydro-3-methyl-2*H*-[1,4]benzoxazine (3.60 g, 19.4 mmol) were added to the resulting solution. The reaction mixture was stirred at room temperature for 48 h, then successively washed with 4N HCl (3 × 10 mL), saturated NaCl solution (5 × 10 mL), 5% NaHCO_3_ solution (3 × 10 mL), and water (3 × 10 mL), dried over MgSO_4_, and evaporated to dryness under reduced pressure. The residue was recrystallized from a EtOH–water (2:1) mixture.

*1,10-bis((S)-7,8-Difluoro-3-methyl-2H-benzo[b]*[1,4]*oxazin-4(3H)-yl)decan-1,10-dione ((S)-**9**).* Yield 2.87 g (54%), yellowish powder m.p. 41–42 °C. HPLC ((S,S)-Whelk-O1, MeOH–H_2_O, 85:15): *t* 17.3 min. ^1^H NMR (500 MHz, DMSO-*d*_6_) δ (ppm): 1.11 (d, *J* = 6.2 Hz, 6H, 2×Me), 1.27 (br. s, 8H, 4×CH_2_), 1.51–1.58 (m, 4H, 2×CH_2_), 2.42–2.48 (m, 2H, CH_2_), 2.60–2.66 (m, 2H, CH_2_), 4.14 (br. d, *J* = 10.4 Hz, 2H, 2×H-2A benzoxazine), 4.36 (d, *J* = 10.8 Hz, 2H, 2×H-2B benzoxazine), 4.71 (br. s, 2H, 2×H-3 benzoxazine), 6.92 (td, *J* = 9.4, 9.0 Hz, 2H, 2×H-6 benzoxazine), 7.65 (br. s, 2H, 2×H-5 benzoxazine). ^19^F NMR (470 MHz, DMSO-*d*_6_, 100 °C) δ (ppm): 1.86 (br. s, F-8), 19.56 (br. s, F-7). ^13^C NMR (125 MHz, DMSO-*d*_6_) δ (ppm): 15.1, 24.6, 28.5, 28.7, 33.4, 44.8 (br. s), 69.8, 106.8 (d, *J =* 18.0 Hz), 119.3, 121.8, 135.7 (dd, *J =* 9.5, 2.5 Hz), 138.9 (dd, *J =* 243.5, 15.5 Hz), 146.4 (d, *J =* 243.5 Hz), 171.0. Calcd (%) for C_28_H_32_F_4_N_2_O_4_: C 62.68, H 6.01, F 14.16, N 5.22. Found (%): C 62.47, H 6.15, F 14.16, N 5.44.

*1,10-bis(-7,8-Difluoro-3-methyl-2H-benzo[b]*[1,4]*oxazin-4(3H)-yl)decan-1,10-dione (mixture **9**).* Yield 2.61 g (49%), colorless powder m.p. 103–104 °C (EtOH-H_2_O). HPLC ((S,S)-Whelk-O1, MeOH–H_2_O, 85:15): *t_S,S_* 17.6 min (27%), t_R*,S*_ 24.2 min (47%), *t_R,R_* 34.0 min (26%). ^1^H NMR (500 MHz, DMSO-*d*_6_) δ (ppm): 1.11 (d, *J* = 6.1 Hz, 6H, 2×Me), 1.27 (br. s, 8H, 4×CH_2_), 1.49–1.60 (m, 4H, 2×CH_2_), 2.42–2.48 (m, 2H, CH_2_), 2.60–2.66 (m, 2H, CH_2_), 4.14 (br. d, *J* = 10.0 Hz, 2H, 2×H-2A benzoxazine), 4.36 (d, *J* = 10.8 Hz, 2H, 2×H-2B benzoxazine), 4.71 (br. s, 2H, 2×H-3 benzoxazine), 6.92 (td, *J* = 9.6, 8.9 Hz, 2H, 2×H-6 benzoxazine), 7.66 (br. s, 2H, 2×H-5 benzoxazine). ^19^F NMR (470 MHz, DMSO-*d*_6_, 100 °C) δ (ppm): 1.84 (br. s, F-8), 19.40 (br. s, F-7). ^13^C NMR (125 MHz, DMSO-*d*_6_) δ (ppm): 15.1, 24.6, 28.5, 28.6, 33.4, 44.9 (br. s), 69.8, 106.7 (d, *J =* 18.0 Hz), 119.2, 121.8, 135.7 (dd, *J =* 9.5, 2.5 Hz), 138.9 (dd, *J =* 243.5, 15.5 Hz), 146.4 (d, *J =* 242.0 Hz), 171.0. Calcd (%) for C_28_H_32_F_4_N_2_O_4_: C 62.68, H 6.01, F 14.16, N 5.22. Found (%): C 62.76, H 6.15, F 14.03, N 5.25.

### 3.2. Cytotoxicity Assessment

#### 3.2.1. Materials

The following materials were used: dimethyl sulfoxide (DMSO, PanEco, Moscow, Russia), DMEM/F12 (Gibco, Grand Island, NY, USA), RPMI-1640 (Gibco, Grand Island, NY, USA), 100 × Glutamax (Gibco, Grand Island, NY, USA), fetal bovine serum (FBS, Gibco, Grand Island, NY, USA), HEPES (Sigma-Aldrich, St. Louis, MO, USA), PBS (Sigma-Aldrich, St. Louis, MO, USA), propidium iodide (Sigma-Aldrich, St. Louis, MO, USA), RNAse A (Sigma-Aldrich, St. Louis, MO, USA), Triton X-100 (PanReac AppliChem, Barcelona, Spain), 100 × Penicillin-Streptomycin (PanEco, Moscow, Russia), and L-glutamine (PanEco, Moscow, Russia). TrypLE (Gibco, Grand Island, NY, USA) was used for dissociating adherent cells from the surface of plastic flasks.

#### 3.2.2. Cell Lines

The following cell lines were used: CT-26 murine colon carcinoma (ATCC), 4T1 murine mammary carcinoma (ATCC), MDA-MB-231 human breast adenocarcinoma (ECACC), COLO201 human colorectal adenocarcinoma (ATCC), HepG2 human hepatocellular carcinoma (State Research Center of Virology and Biotechnology “Vector”, Koltsovo, Russia); A549 human non-small-cell lung carcinoma, SK-BR-3 human breast adenocarcinoma, SNU-1 human gastric carcinoma, Jurkat human acute T-lymphoblastic leukemia, and WI-38 human lung fibroblasts (normal cells) obtained from the Bioresource Collection of Cell Lines and Primary Tumors of the Blokhin National Medical Research Center of Oncology (Moscow, Russia). Cells were counted on a Countess^®^ II FL automatic cell counter (Thermo Fisher Scientific, Singapore).

#### 3.2.3. MTT Cytotoxicity Assay

Cells were seeded in flat-bottomed 96-well plates at 10,000 cells per well (for A549, SNU-1, WI-38, CT-26, MDA-MB-231, 4T1, and HepG2 cells) or 20,000 cells per well (for SK-BR-3, Jurkat, and COLO201 cells) and cultivated in complete culture mediums (for composition of each medium, see the Supplementary Materials, Table S1) in a CO_2_ incubator at 37 °C. At the first stage of this study, the test compounds dissolved in DMSO were added to the wells with cells at final concentrations from 1 × 10^–4^ to 1 × 10^–10^ mol/L (A549, SK-BR-3, SNU-1, Jurkat, and WI-38) or 1 × 10^–3^ to 1 × 10^–8^ mol/L (CT-26, MDA-MB-231, 4T1, COLO201, and HepG2); DMSO concentration in the wells was finally 1%. A complete cultural medium and DMSO were added to the control wells at the same concentration as in the experimental wells; 0.1% Triton X-100 solution (10 µL) was added to the corresponding wells as a non-specific positive control. After adding the test compounds, the cells were incubated for 72 h in an atmosphere containing 5% CO_2_. After that, the nutrient medium was removed from each well (in the case of SK-BR-3, Jurkat, and COLO201, plates were pre-centrifuged at 1500 rpm for 6 min). A solution of the commercial anticancer agent Doxorubicin (Dox, Sigma-Aldrich, St. Louis, MO, USA) in DMSO was used as a reference drug. Then, 150 µL of a complete medium with the MTT reagent (PanEco, Moscow, Russia) at a concentration of 0.5 mg/mL was added and incubated for 2 h. Formazan crystals were dissolved in DMSO (200 µL) and the absorbance in the wells was measured at 540 nm and a reference wavelength of 650 nm (Sunrise, Tecan, Groding, Austria). Experiments were performed in three parallel runs. Cell viability after incubation in the presence of test compounds was calculated in relation to cell viability in the control wells according to the Formula (1):

Cell viability (%) = (*A*_exp_/*A*_c_) × 100, (1)

where *A*_exp_ is the optical density of solutions in the wells containing test compounds; *A*_c_ is the optical density of solutions in control wells without test compounds. For detailed information on cell viability of various cell lines, see the Supplementary Materials, Figures S21–S26.

At the second stage of this study, in order to more accurately calculate the CC_50_ (the half maximal inhibitory concentration), the cytotoxic effect of test compounds was evaluated at concentrations close to CC_50_ found at the first stage. The study of the cytotoxic activity of each substance towards the cells of each line was carried out in triplicate; the mean value was used to calculate CC_50_. The CC_50_ values were determined by non-linear regression analysis using Graph-Pad Prism7.

#### 3.2.4. Cell Cycle Analysis

Cell cycle analysis was performed by flow cytometry by staining DNA with propidium iodide (PI). For this, cells were seeded into 6-well plates: 2 × 10^5^ COLO201 cells per well and 1.2 × 10^5^ MDA-MB-231 cells per well. The next day, a solution of compound **1d** in DMSO at a concentration of 1 × 10^–2^ mol/L was prepared, and then a series of sequential dilutions was obtained; for each cell line, compound **1d** was tested at three concentrations close to CC_50_. The final concentration of DMSO in the culture medium was 0.1%; an equivalent volume was added to the control cells. The plates were incubated for 24 h in a CO_2_ incubator. Then, cells were detached from the plastic surface using TrypLE, washed with PBS, and fixed with 70% ethanol. Next, the cells were incubated for 1 h in the dark at 4 °C, then sedimented by centrifugation at 184× *g* for 10 min, and washed twice with PBS. PBS (0.3 mL) containing 0.1% Triton X-100, 1 µg/mL PI, and 0.2 mg/mL RNAse A was added to the pellet, incubated for 30 min at room temperature in the dark, and analyzed on a CytoFlex flow cytometer (Beckman Coulter, Brea, CA, USA).

The cell population in the test sample was found and gated on forward versus side scatter (FSC-A vs. SSC-A). Next, doublets were discriminated and removed via PI signal height versus PI signal area (PI-H vs. PI-A). For cell cycle phases analysis, PI-A was plotted against number of events (number of cells) (PI-A vs. Count).

The flow cytometry data were processed using the Kaluza Analysis 2.1 software (Beckman Coulter, Brea, CA, USA). Percentages of cell populations distributed in the various phases of the cell cycle (sub-G1, G0/G1, S, and G2M) were calculated. The result was considered significant if the coefficient of variation (CV) was less than 6.

Representative diagrams of the COLO201 and MDA-MB-231 cell distribution by cell cycle phases are presented in the Supplementary Materials (Figures S27 and S28).

#### 3.2.5. Statistical Analysis

Statistical data processing was carried out using GraphPad Prism 5.0, GraphPad Prism 7 (GraphPad Software, San Diego, CA, USA), and Microsoft Office Excel software packages. Descriptive statistics were used for all data. The normality of data distribution was tested using the Shapiro–Wilks test. Data were presented as the mean ± standard deviation (M ± SD). To identify the significance of differences in the case of multiple comparisons, one-way analysis of variance (one-way ANOVA) was used for group comparison, then the Tukey’s test for pairwise comparisons between groups. Differences were considered significant at *p* ˂ 0.05.

## 4. Conclusions

In summary, the high cytotoxic activity of purinyl derivatives of omega-aminoalkanoyl-3,4-dihydro-3-methyl-7,8-difluoro-2*H*-[1,4]benzoxazine, especially compound **1d**, against A549, SK-BR-3, SNU-1, Jurkat, CT-26, MDA-MB-231, 4T1, COLO201, and HepG2 tumor cells has been found. Among the cell lines studied, 4T1 murine breast carcinoma, COLO201 human colorectal adenocarcinoma, SNU-1 gastric cancer, and HepG2 human hepatocellular carcinoma cells showed the highest sensitivity. The cytotoxicity against these tumor cells in experiments exceeded the cytotoxicity against normal cells (human lung fibroblasts WI-38); selective cytotoxicity index reached 145.

We have obtained structural analogs of the most active compounds; the study of their cytotoxicity made it possible to find that the presence of both a purine fragment and difluorobenzoxazine bound via a polymethylene linker of a certain length is important for the manifestation of cytotoxic activity of this group of compounds. The introduction of a 2-hydroxyethoxymethyl group into the molecule of compound **1d** resulted in a compound that was not inferior to it in terms of cytotoxic activity.

The study of the effect of the most promising compound **1d** on the cell cycle of two types of human tumor cell lines, the most highly sensitive and the least sensitive to cytotoxic action, allows us to conclude that this compound is a blocker of DNA biosynthesis. Thus, we have found a group of potential antitumor agents, purine conjugates with high cytotoxicity against tumor cell lines.

## Figures and Tables

**Table 1:** CC_50_ (µM) and SCI values for compounds **1c–f** after incubation with cells for 72 h (MTT assay, *n* = 3).

Cell Line	Compound							
	1c		1d		1e		1f	
	CC_50_	SCI	CC_50_	SCI	CC_50_	SCI	CC_50_	SCI
WI-38	50	-	21	-	71	-	35	-
CT-26	19	2.6	19	1.1	3.4	21	3.0	12
4T1	23	2.2	23	0.91	0.49	145	2.3	15
MDA-MB-231	44	1.2	44	0.48	12	5.9	28	1.4
COLO201	0.68	73	0.68	31	3.5	20	1.3	27
HepG2	8.5	5.9	8.5	2.5	5.2	14	9.7	3.6
A549	43	1.2	43	0.49	75	0.95	60	0.58
SK-BR-3	75	0.67	75	0.28	85	0.83	99	0.35
SNU-1	7.0	7.1	7.0	3.0	6.0	12	6.0	5.8
Jurkat	8.5	5.9	8.5	2.5	4.0	18	32	1.1

SCI values were calculated dividing the CC_50_ of WI-38 normal cell line by the CC_50_ of the cancer cell line for each compound.

**Table 2:** Cell viability (%) of compounds **1c–e** at a concentration of 1 × 10^–4^ M. Data are presented as the mean ± SD (*n* = 3).

Compound	CT-26	4T1	MDA-MB-231	COLO201	HepG2	A549	SK-BR-3	SNU-1	Jurkat	WI-38
**1c**	2 ± 1	2 ± 2	5 ± 5	2 ± 1	2 ± 1	7 ± 1	8 ± 0	6 ± 2	8 ± 2	21 ± 3
**1d**	2 ± 1	2 ± 1	4 ± 2	1 ± 1	2 ± 1	6 ± 1	8 ± 2	5 ± 1	10 ± 4	21 ± 7
**1e**	3 ± 2	2 ± 2	7 ± 5	2 ± 1	2 ± 1	6 ± 0	9 ± 2	5 ± 1	10 ± 4	22 ± 7

**Table 3:** CC_50_ (M) and SCI values for studied compounds **1a–d**, **2a–c**, **6**, **7**, and **9** after incubation with cells for 72 h (MTT assay, *n* = 3).

Compound	Cell Lines								
	WI-38	A549		SK-BR-3		SNU-1		Jurkat	
	CC_50_	CC_50_	SCI	CC_50_	SCI	CC_50_	SCI	CC_50_	SCI
**1a**	>1.0 × 10^–4^	>1.0 × 10^–4^	1.0	>1.0 × 10^–4^	1.0	>1.0 × 10^–4^	1.0	>1.0 × 10^–4^	1.0
**1b**	9.8 × 10^–5^	5.3 × 10^–5^	1.8	>1.0 × 10^–4^	0.98	2.0 × 10^–5^	4.9	6.7 × 10^–5^	1.5
(*S*)-**1b**	8.1 × 10^–5^	5.8 × 10^–4^	0.14	>1.0 × 10^–4^	0.81	2.6 × 10^–5^	3.1	3.7 × 10^–5^	2.2
**1d**	2.1 × 10^–5^	2.1 × 10^–5^	1.0	2.8 × 10^–5^	0.75	2.0 × 10^–6^	10	1.0 × 10^–5^	2.1
**2a**	>1.0 × 10^–4^	>1.0 × 10^–4^	1.0	>1.0 × 10^–4^	1.0	>1.0 × 10^–4^	1.0	5.9 × 10^–5^	1.7
**2b**	>1.0 × 10^–4^	>1.0 × 10^–4^	1.0	>1.0 × 10^–4^	1.0	>1.0 × 10^–4^	1.0	4.9 × 10^–5^	2.0
**2c**	>1.0 × 10^–4^	>1.0 × 10^–4^	1.0	>1.0 × 10^–4^	1.0	>1.0 × 10^–4^	1.0	>1.0 × 10^–4^	1.0
**6**	3.0 × 10^–5^	1.5 × 10^–5^	2.0	3.7 × 10^–4^	0.08	5.6 × 10^–6^	5.4	1.0 × 10^–5^	3.0
**7**	1.4 × 10^–5^	9.4 × 10^–6^	1.5	4.2 × 10^–5^	0.33	3.7 × 10^–6^	3.8	7.3 × 10^–6^	1.9
(*S*)-**9**	>1.0 × 10^–4^	>1.0 × 10^–4^	1.0	>1.0 × 10^–4^	1.0	>1.0 × 10^–4^	1.0	>1.0 × 10^–4^	1.0
**9**	>1.0 × 10^–4^	>1.0 × 10^–4^	1.0	>1.0 × 10^–4^	1.0	>1.0 × 10^–4^	1.0	>1.0 × 10^–4^	1.0
Dox	2.3 × 10^–7^	2.2 × 10^–7^	1.0	1.5 × 10^–6^	0.15	1.4 × 10^–7^	1.6	1.2 × 10^–7^	1.9

SCI values were calculated dividing the CC_50_ of WI-38 normal cell line by the CC_50_ of the cancer cell line for each compound.

## Data Availability

Not applicable.

## Supplementary Materials

The following supporting information can be downloaded at: https://www.mdpi.com/article/10.3390/molecules28041853/s1. Figures S1–S18: ^1^H, ^19^F, and ^13^C NMR spectra of compounds **2a–c**, **6**, **7**, (*S*)-**9**, and **9**. Figures S19 and S20: HPLC data for compounds (*S*)-**9** and **9**. Table S1: Cell lines and composition of culture medium. Figure S21: Viability of WI-38, A549, SK-BR-3, SNU-1, Jurkat, CT-26, MDA-MB-231, 4T1, COLO201, HepG2 cells vs. logarithmic concentration of compound **1c** after a 72-h incubation. Figure S22: Viability of WI-38, A549, SK-BR-3, SNU-1, Jurkat, CT-26, MDA-MB-231, 4T1, COLO201, HepG2 cells vs. logarithmic concentration of compound **1d** after a 72-h incubation. Figure S23: Viability of WI-38, A549, SK-BR-3, SNU-1, Jurkat, CT-26, MDA-MB-231, 4T1, COLO201, HepG2 cells vs. logarithmic concentration of compound **1e** after a 72-h incubation. Figure S24: Viability of WI-38, A549, SK-BR-3, SNU-1, Jurkat, CT-26, MDA-MB-231, 4T1, COLO201, HepG2 cells vs. logarithmic concentration of compound **1f** after a 72-h incubation. Figure S25. Viability of WI-38, A549, SK-BR-3, SNU-1, and Jurkat cells vs. logarithmic concentration of compound **6** after a 72-h incubation. Figure S26: Viability of WI-38, A549, SK-BR-3, SNU-1, and Jurkat cells vs. logarithmic concentration of compound **7** after a 72-h incubation. Figure S27: Diagrams of the COLO201 cell distribution by cell cycle phases. Figure S28: Diagrams of the MDA-MB-231 cell distribution by cell cycle phases. Table S2: Effect of compound **1d** on the cell cycle of the COLO201 cells. Table S3: Effect of compound **1d** on the cell cycle of the MDA-MB-231 cells.
