# Supplementary material for: Synthesis and Cytotoxic Activity of the Derivatives of N-(Purin-6-yl)aminopolymethylene Carboxylic Acids and Related Compounds
Source: Molecules. 2023 Feb 15;28(4):1853. doi: 10.3390/molecules28041853 (PMC9962735; doi:10.3390/molecules28041853)
Supplement: Supplementary file 1 [file molecules-28-01853-s001.zip › molecules-2187924-supplementary.pdf]

Supplementary Materials

Synthesis and Cytotoxic Activity of *N*-(Purin-6-yl)aminopolymethylene Carboxylic Acids and Related Compounds

Victor P. Krasnov [1,*], Olga A. Vozdvizhenskaya [1], Maria A. Baryshnikova [2], Alexandra G. Pershina [3,4], Vera V. Musiyak [1], Tatyana V. Matveeva [1], Kseniya V. Nevskaya [3], Olga Ya. Brikunova [3], Dmitry A. Gruzdev [1] and Galina L. Levit [1]

[1] Postovsky Institute of Organic Synthesis, Russian Academy of Sciences (Ural Branch), Ekaterinburg 620108, Russia
[2] Blokhin National Medical Research Center of Oncology, Ministry of Health of the Russian Federation, Moscow 115522, Russia
[3] Center of Bioscience and Bioengineering, Siberian State Medical University, Tomsk 634050, Russia
[4] Research School of Chemical and Biomedical Engineering, National Research Tomsk Polytechnic University, Tomsk 634050, Russia

Table of Contents

NMR Spectra S2

HPLC Data S11

Cytotoxicity Assessment S12

Cell Cycle Analysis S15

NMR Spectra

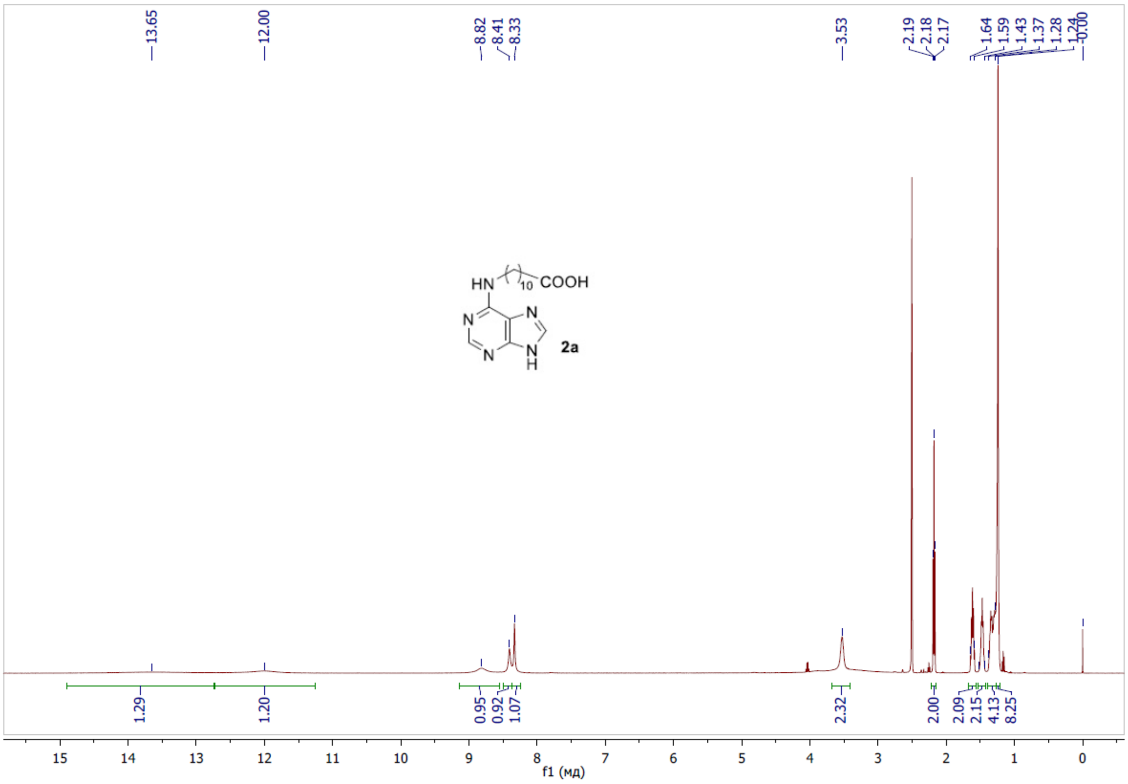

Figure S1. ^{1}H NMR spectrum of compound **2a** (DMSO-d_6, 500 MHz)

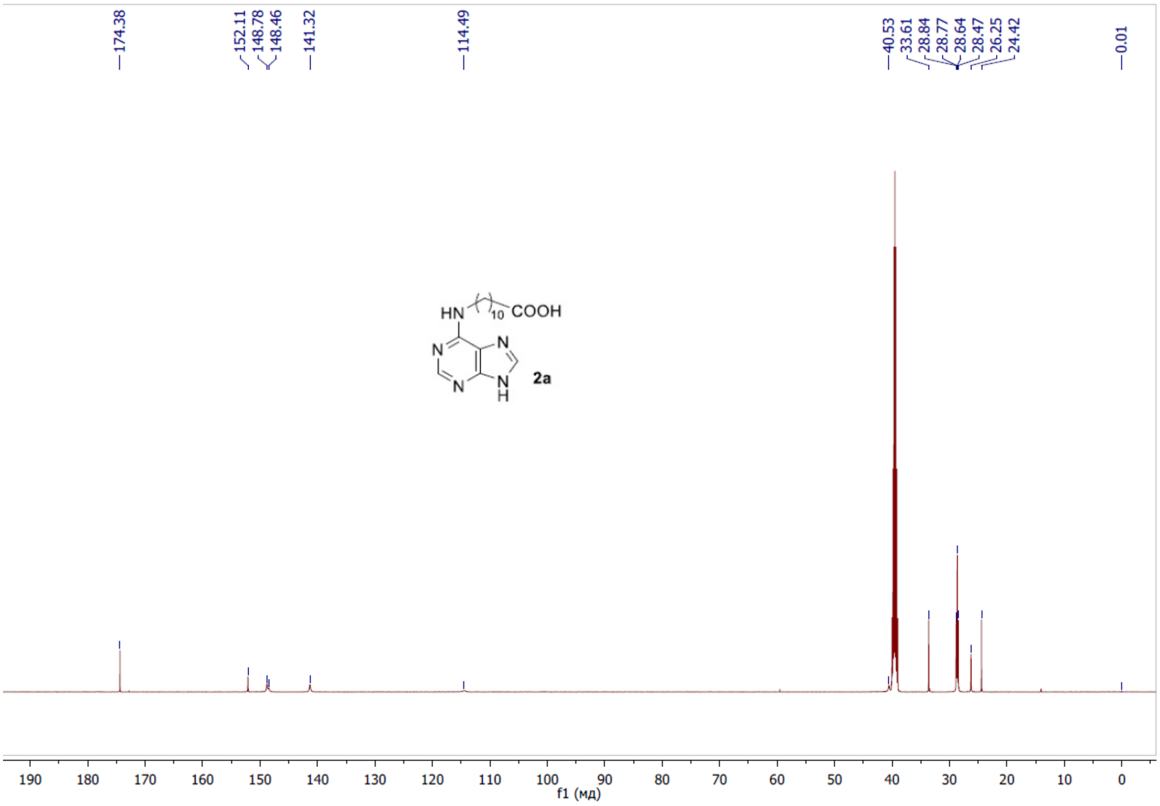

Figure S2. ^{13}C NMR spectrum of compound **2a** (DMSO-d_6, 125 MHz)

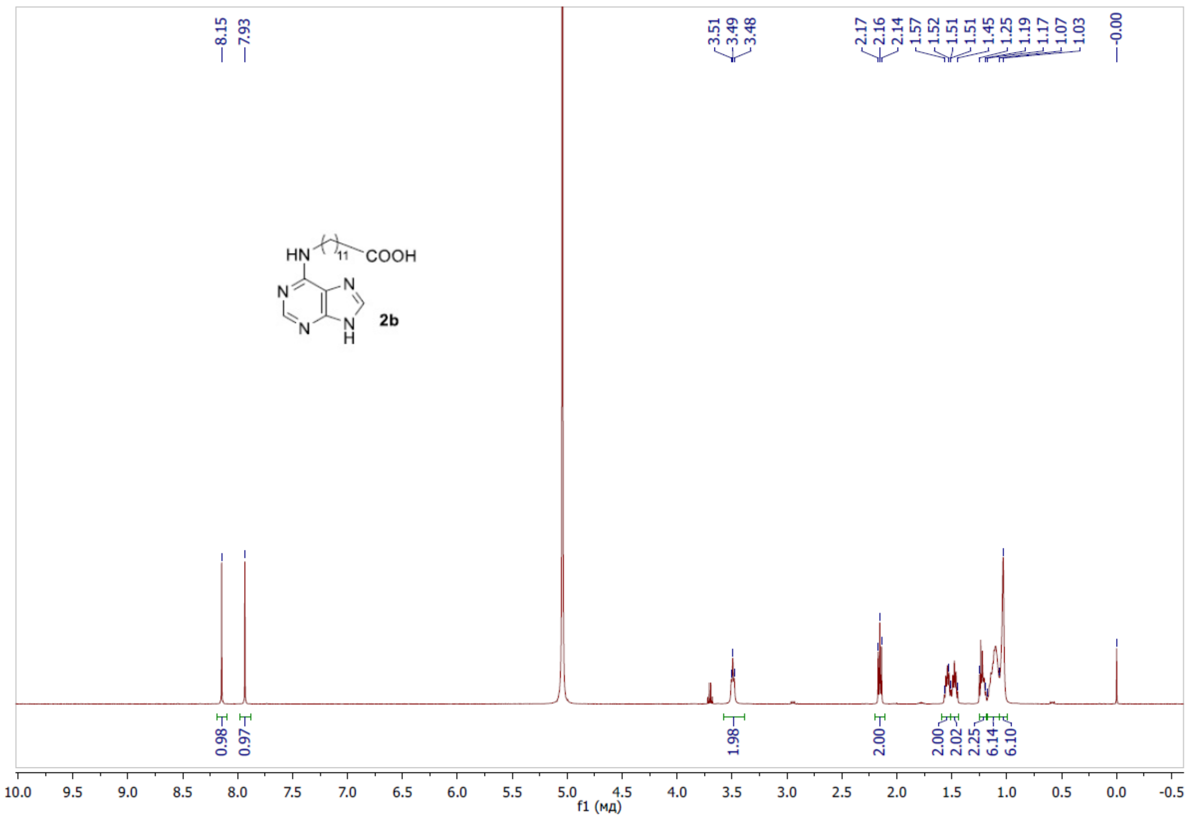

Figure S3. ^{1}H NMR spectrum of compound **2b** (D_2O + NaOD, 500 MHz)

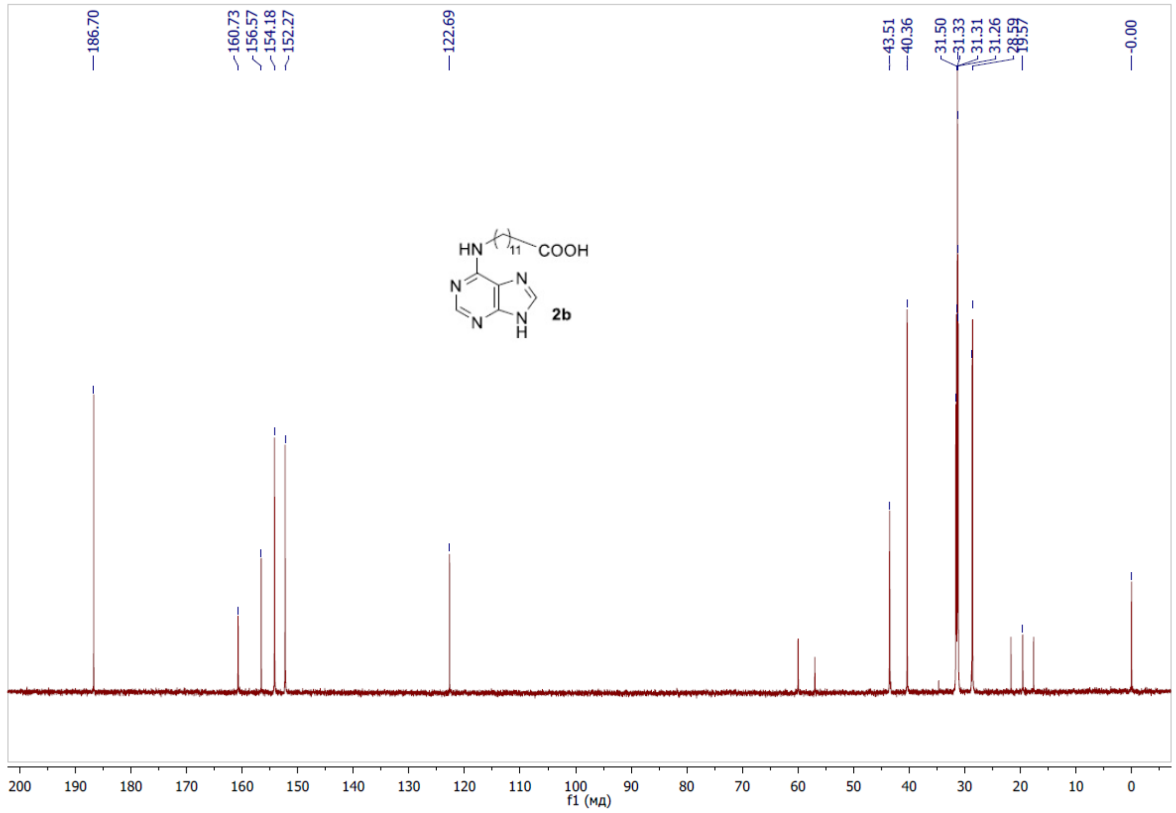

Figure S4. ^{13}C NMR spectrum of compound **2b** (D_2O + NaOD, 125 MHz)

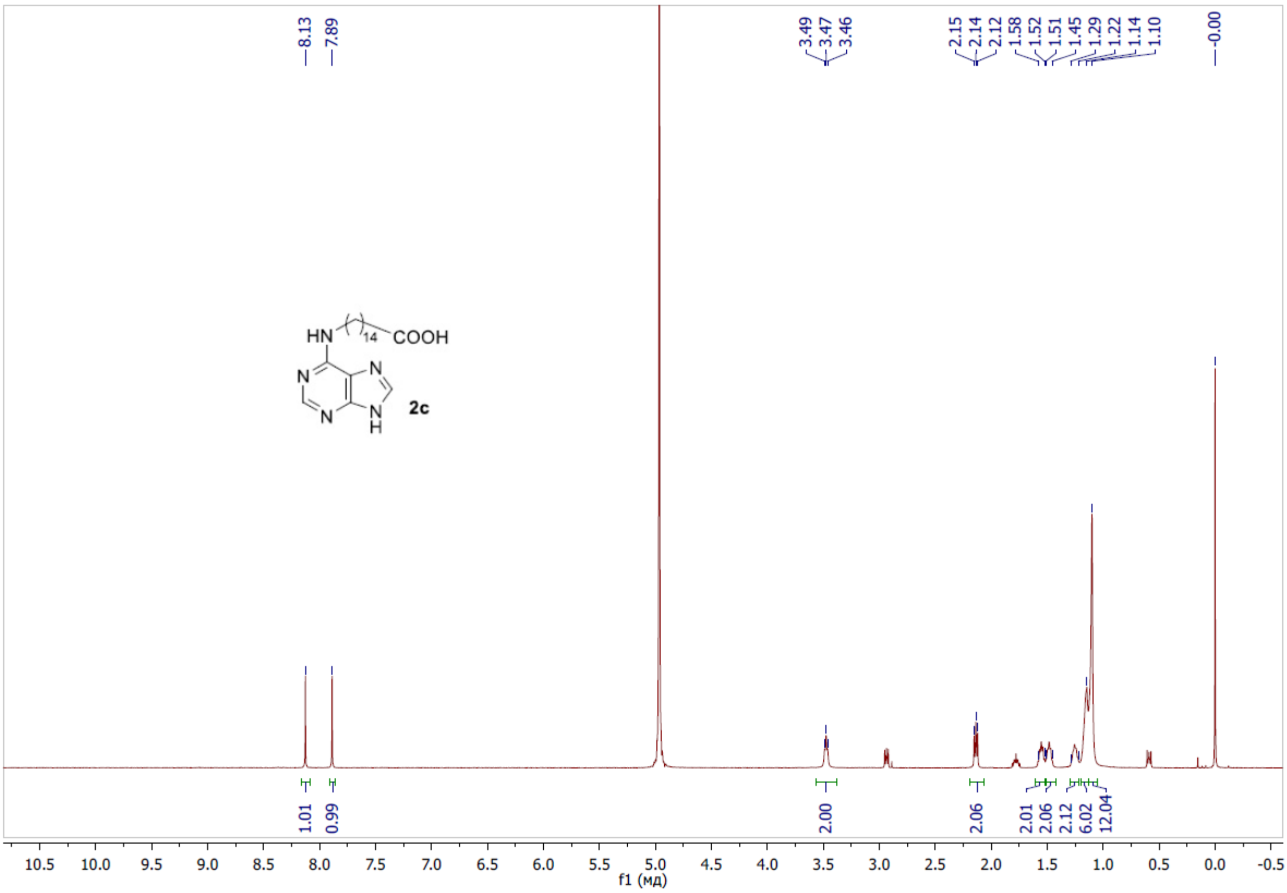

Figure S5. ^{1}H NMR spectrum of compound **2c** (D_2O + NaOD, 500 MHz)

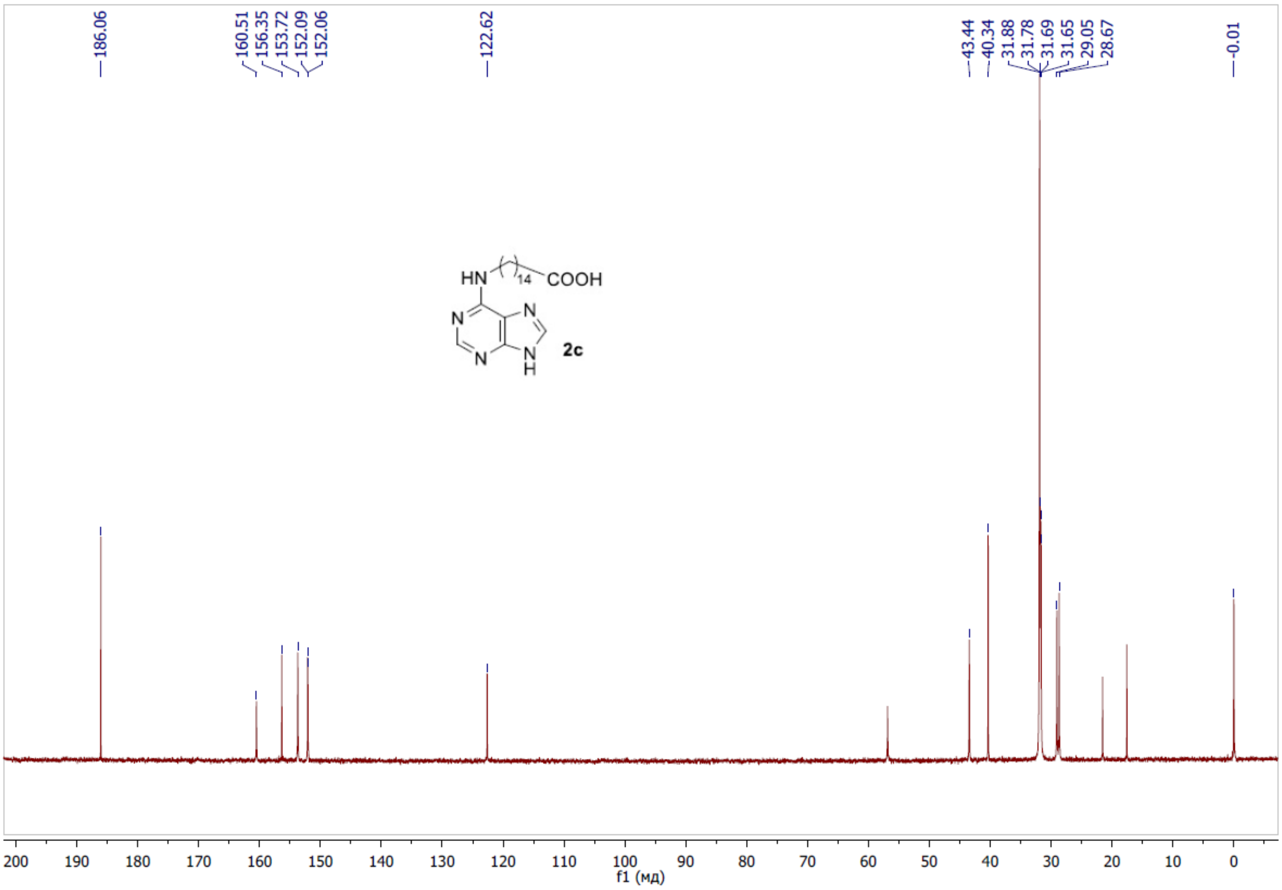

Figure S6. ^{13}C NMR spectrum of compound **2c** (D_2O + NaOD, 125 MHz)

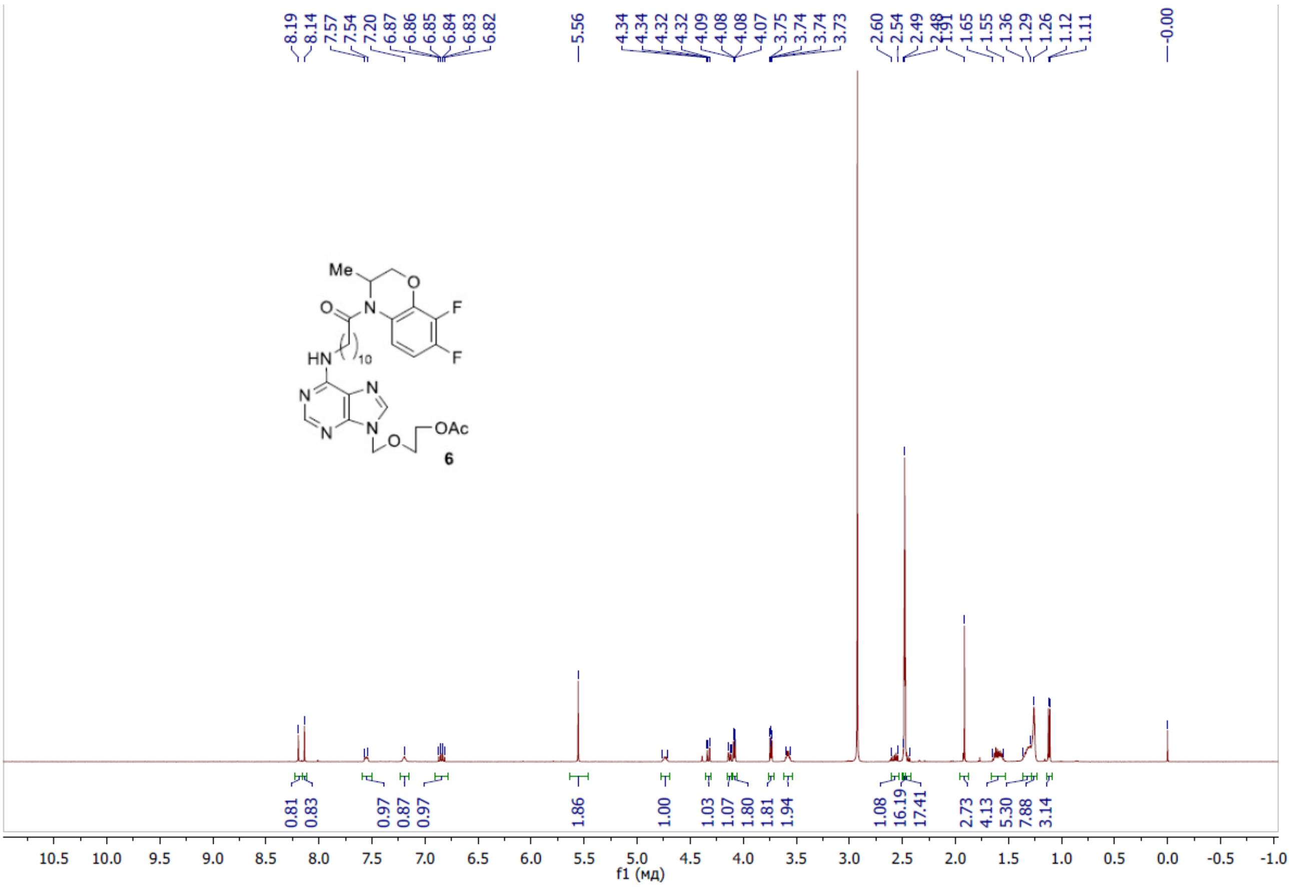

Figure S7. ^{1}H NMR spectrum of compound **6** (DMSO-d_6, 100 °C, 500 MHz)

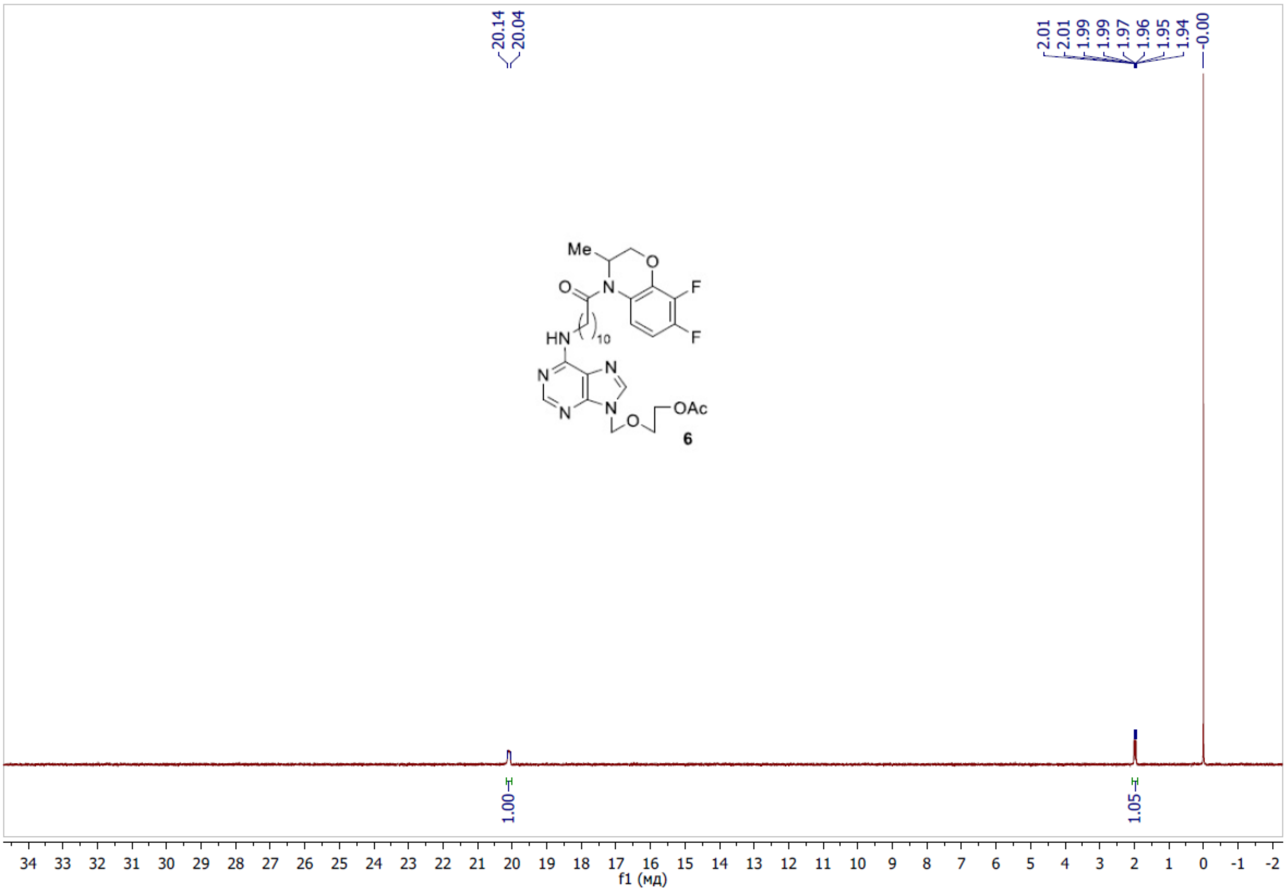

Figure S8. ^{19}F NMR spectrum of compound **6** (DMSO-d_6, 100 °C, 470 MHz)

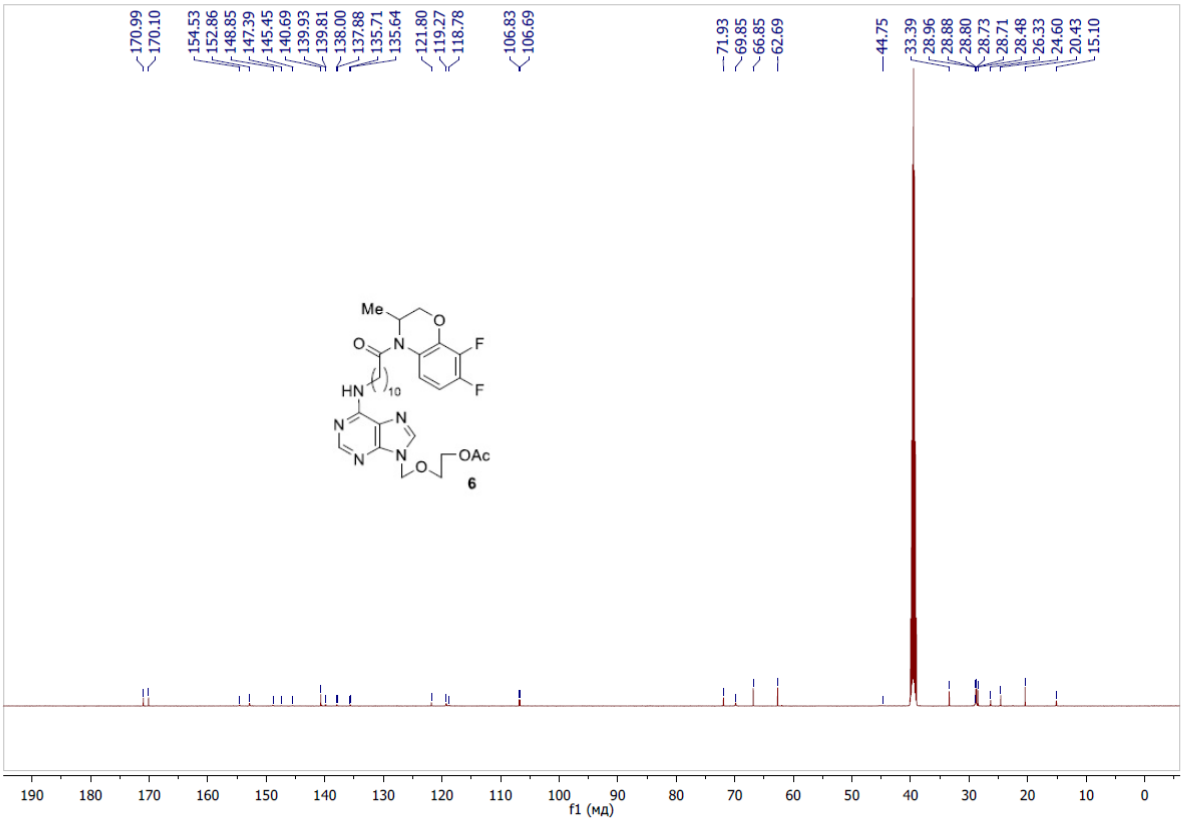

Figure S9. ^{13}C NMR spectrum of compound **6** (DMSO-d_6, 125 MHz)

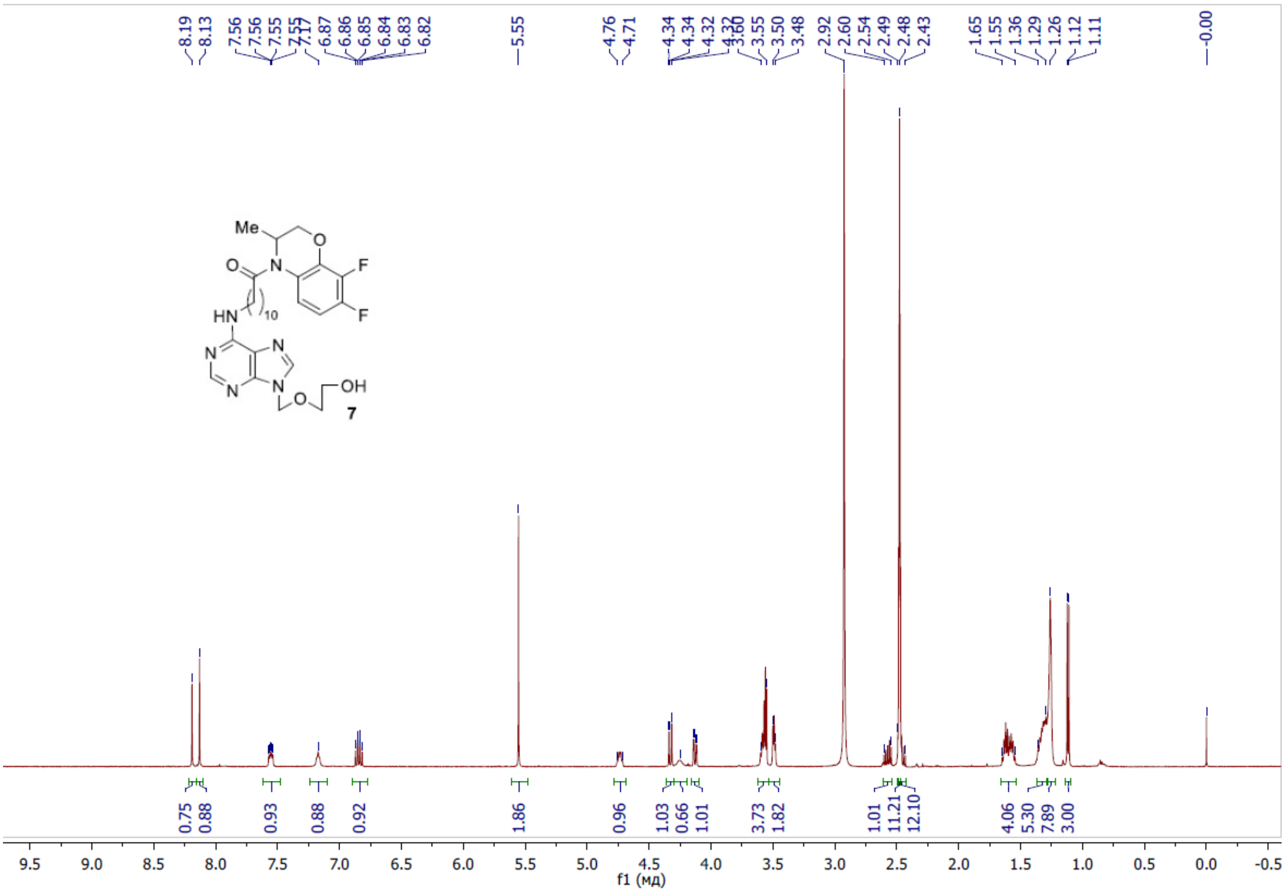

Figure S10. ^{1}H NMR spectrum of compound **7** (DMSO-d_6, 100 °C, 500 MHz)

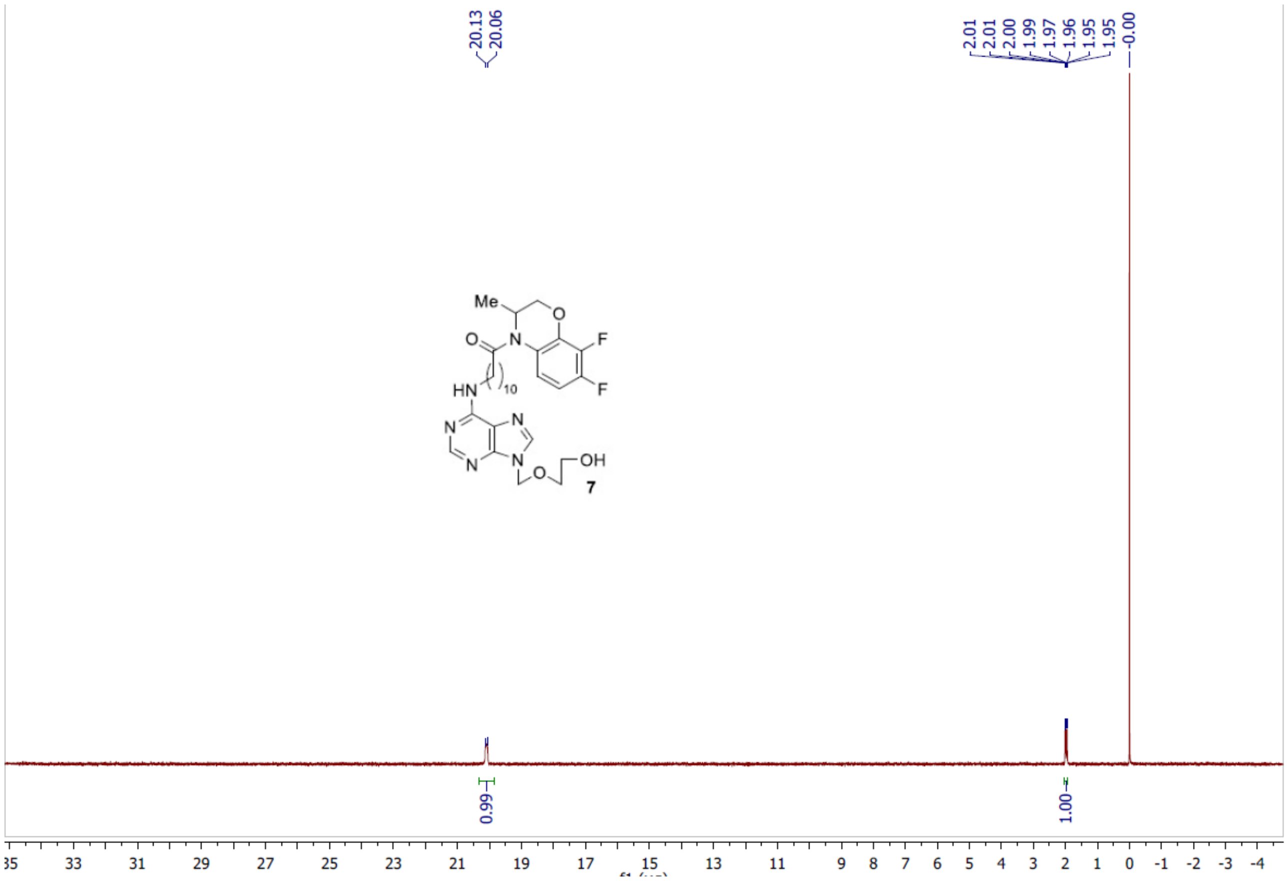

Figure S11. ^{19}F NMR spectrum of compound **7** (DMSO-d_6, 100 °C, 470 MHz)

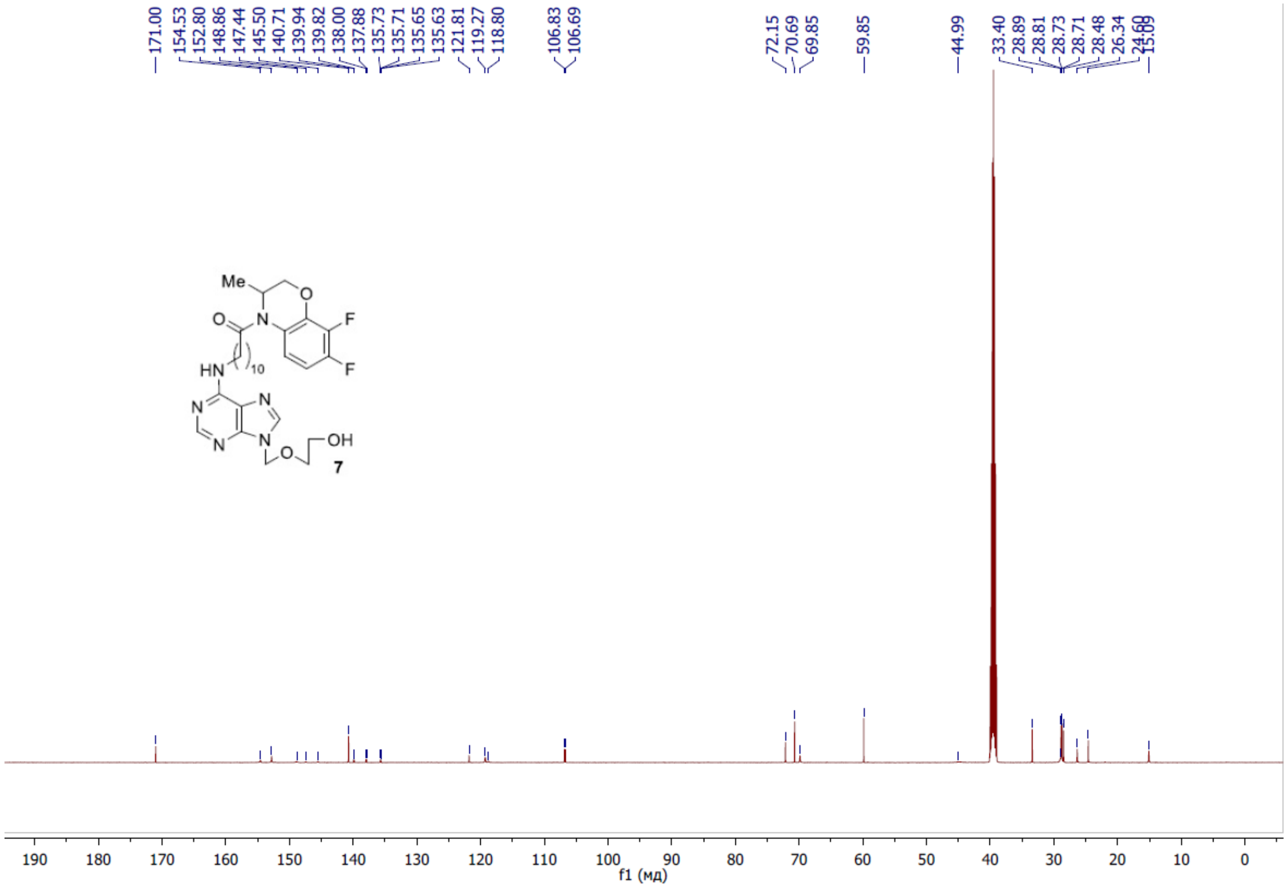

Figure S12. ^{13}C NMR spectrum of compound **7** (DMSO-d_6, 125 MHz)

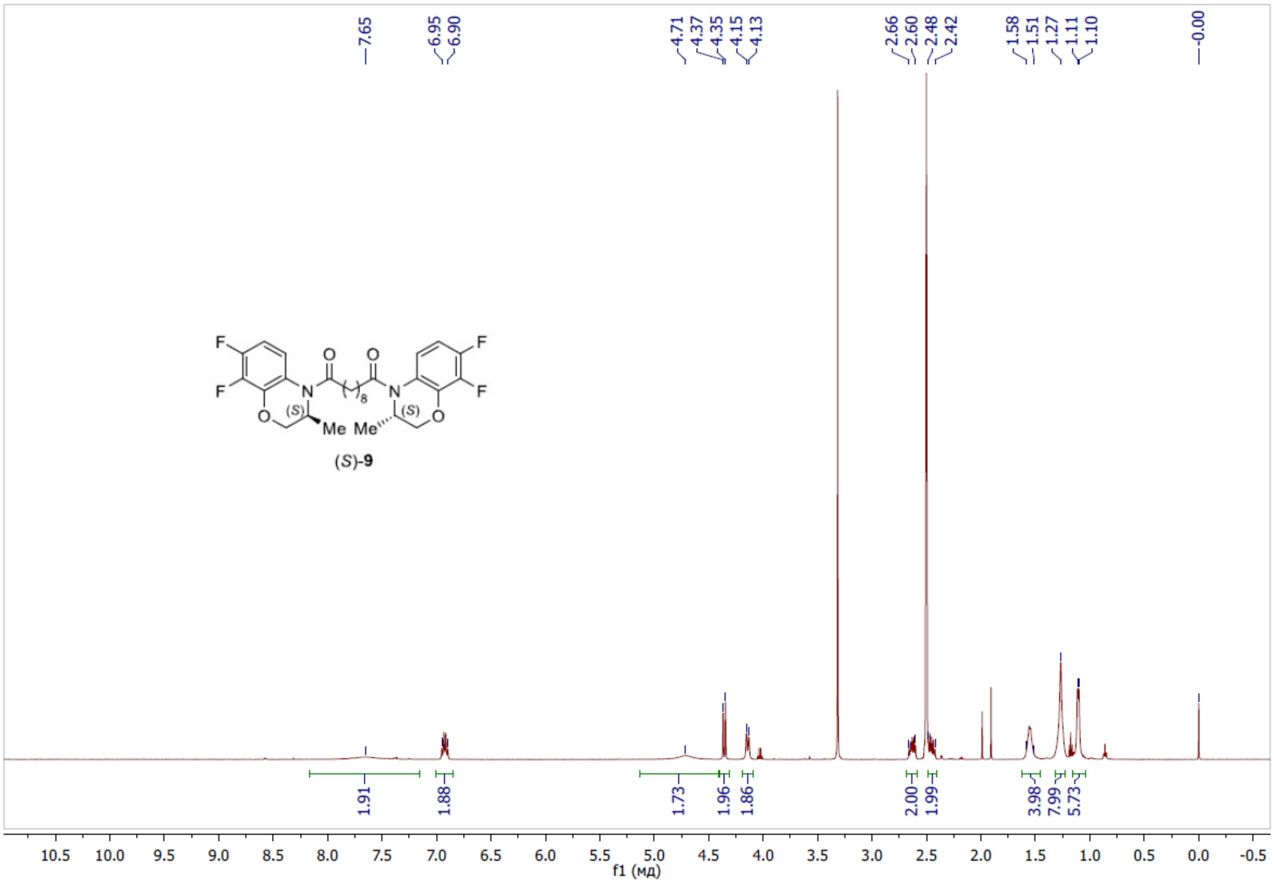

Figure S13. ^{1}H NMR spectrum of compound (*S*)-**9** (DMSO-d_6, 500 MHz)

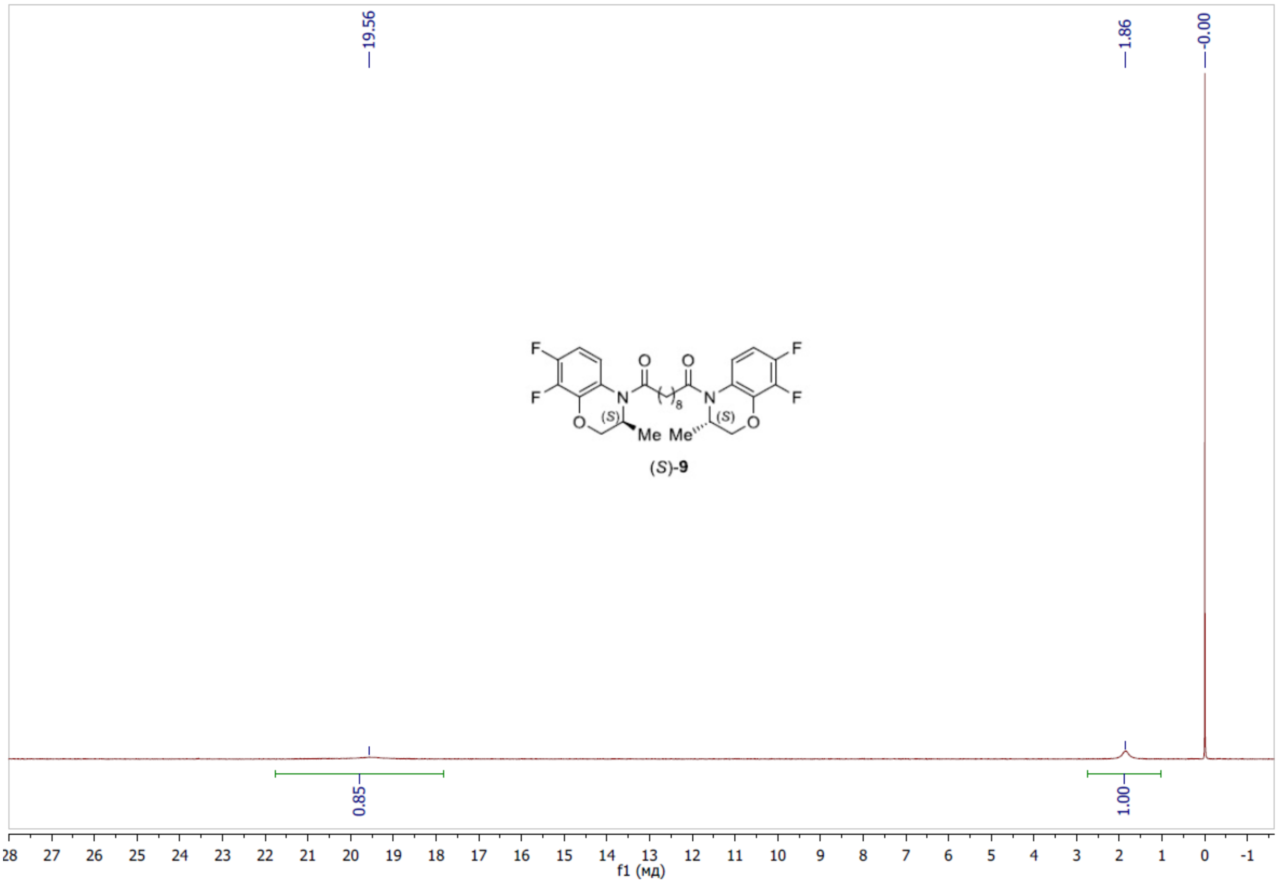

Figure S14. ^{19}F NMR spectrum of compound (*S*)-**9** (DMSO-d_6, 470 MHz)

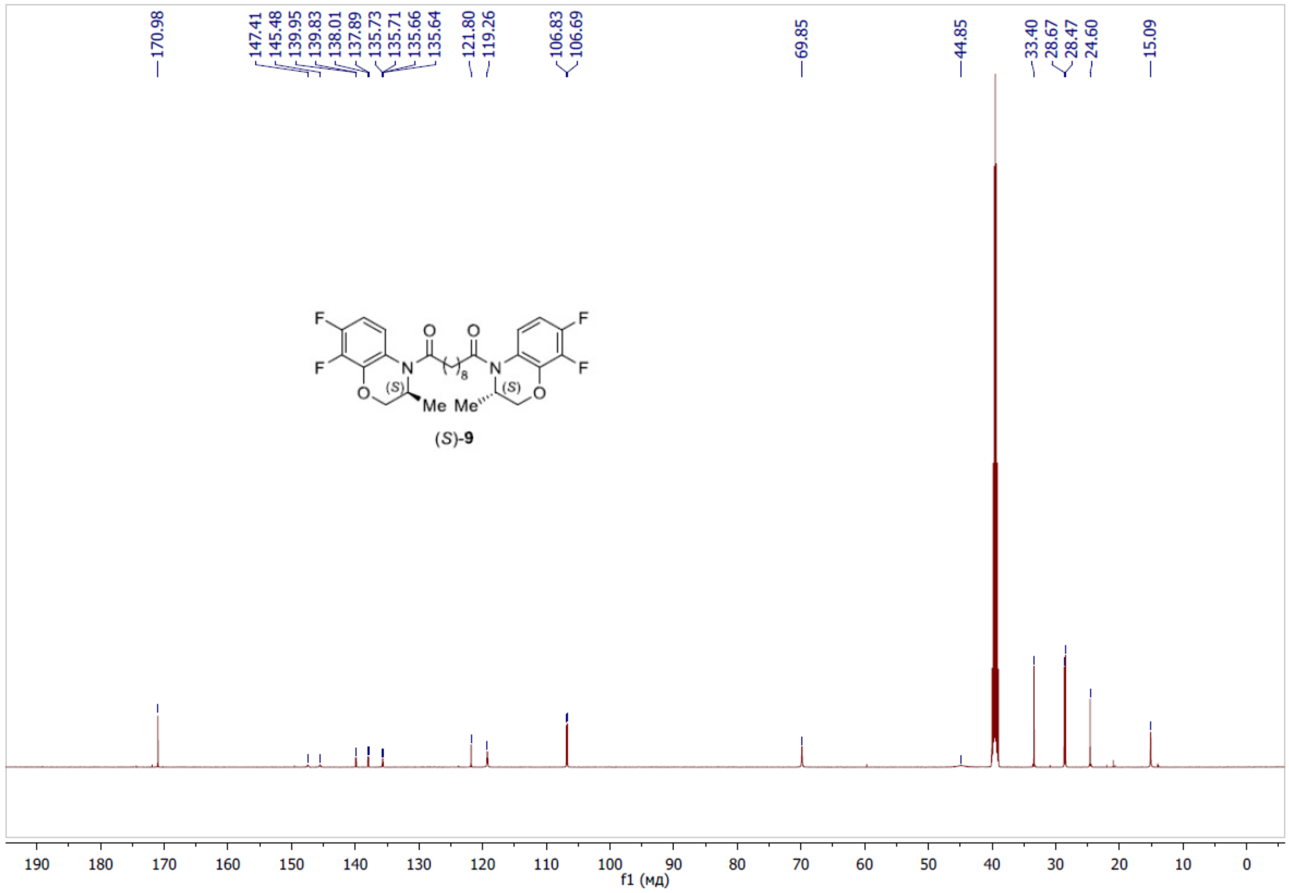

Figure S15. ^{13}C NMR spectrum of compound (*S*)-**9** (DMSO-d_6, 125 MHz)

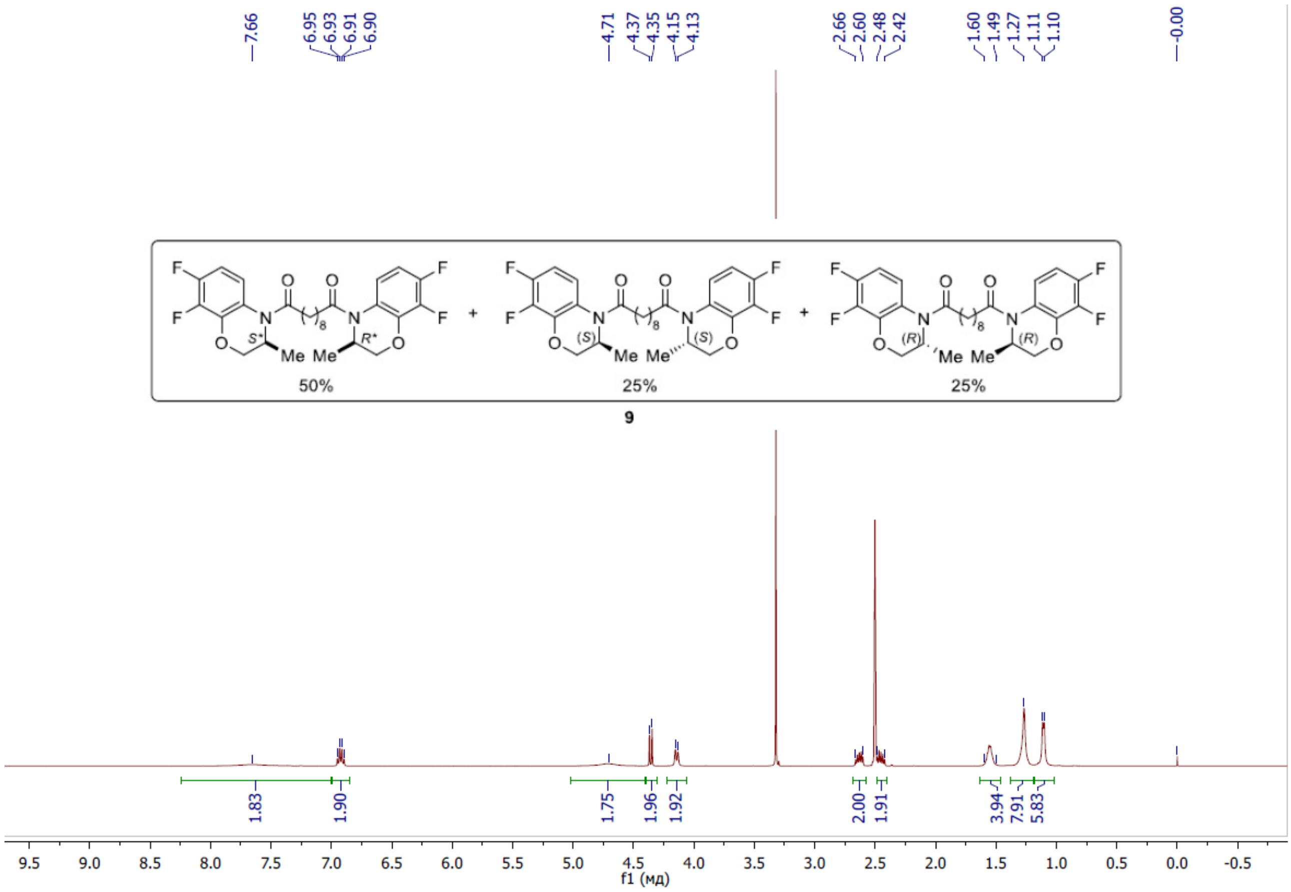

Figure S16. ^{1}H NMR spectrum of compound **9** (DMSO-d_6, 500 MHz)

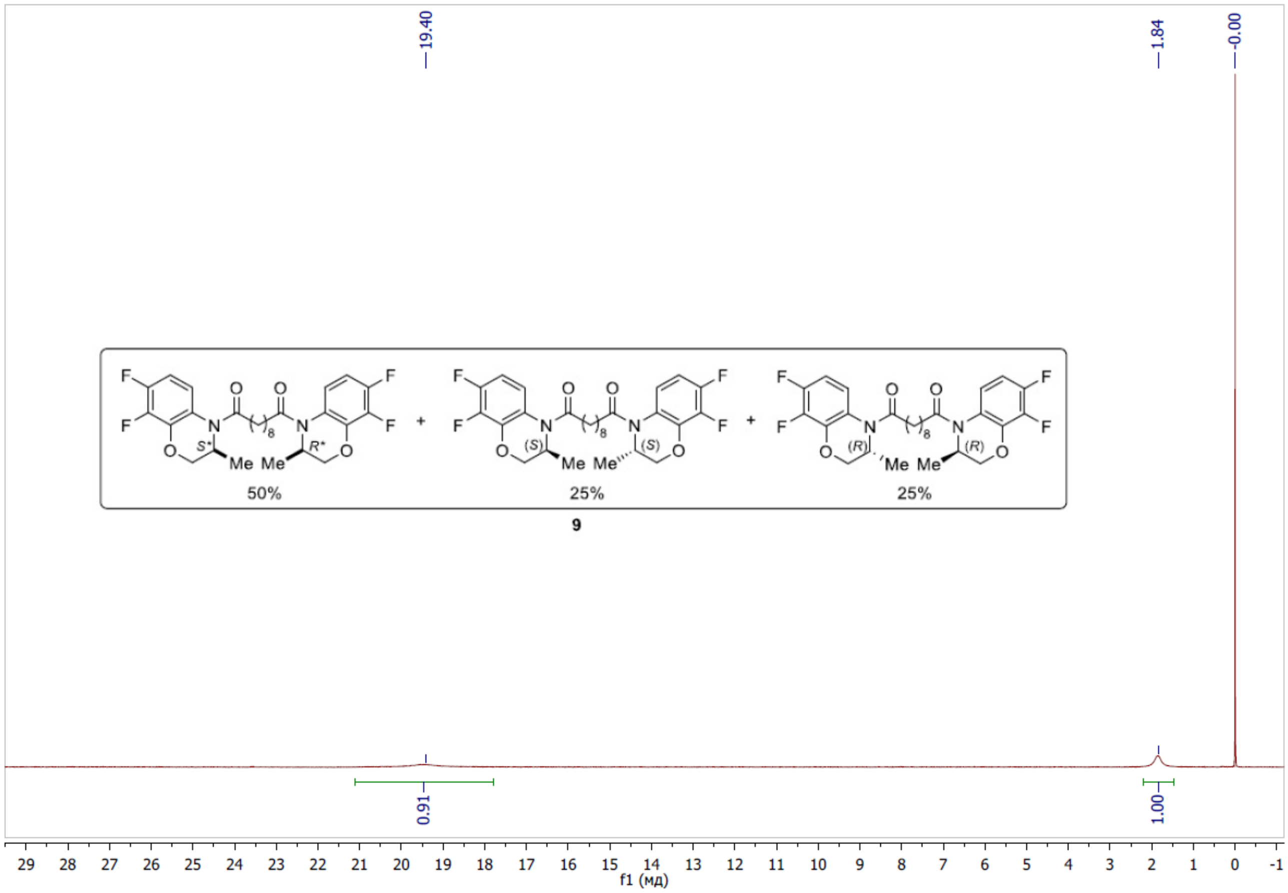

Figure S17. ^{19}F NMR spectrum of compound **9** (DMSO-d_6, 470 MHz)

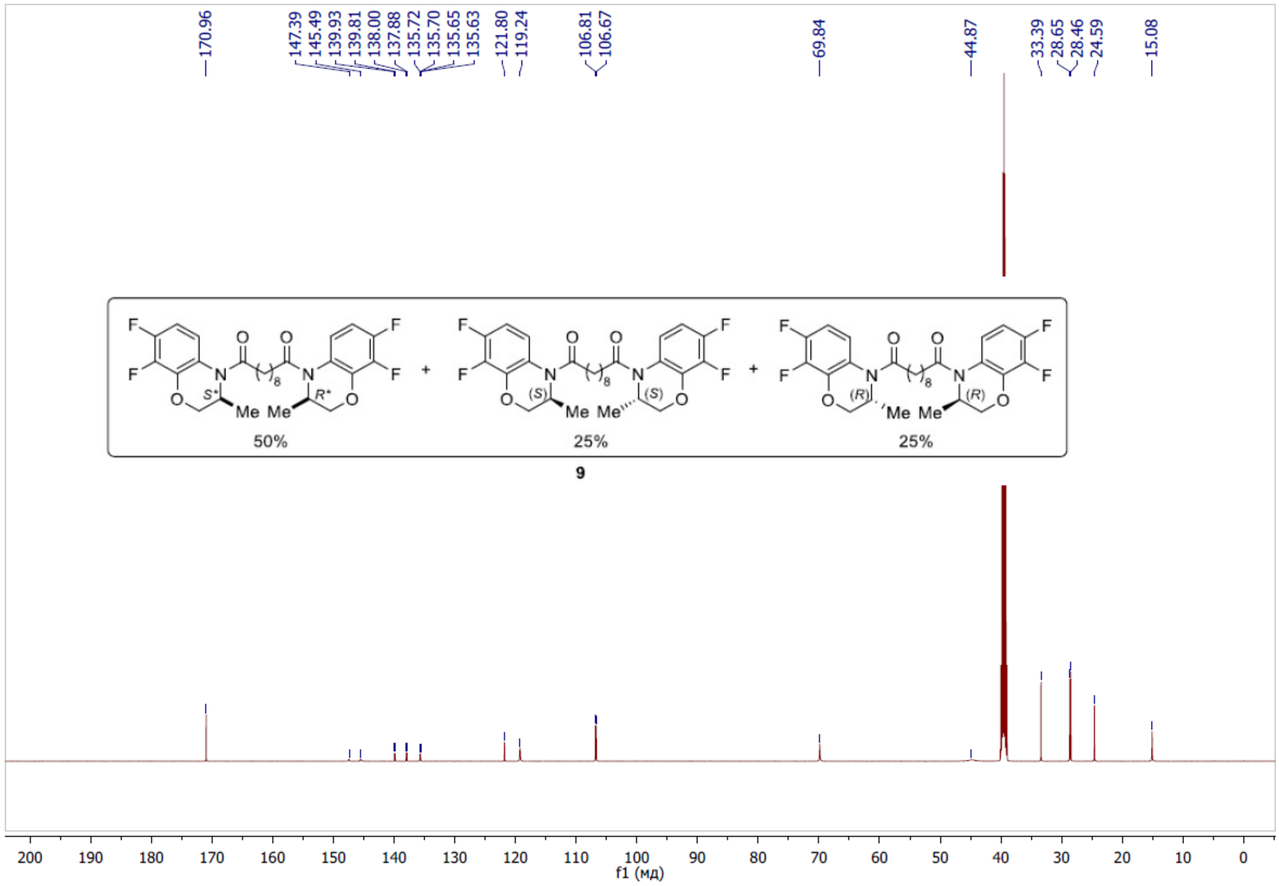

Figure S18. ^{13}C NMR spectrum of compound **9** (DMSO-d_6, 125 MHz)

HPLC Data

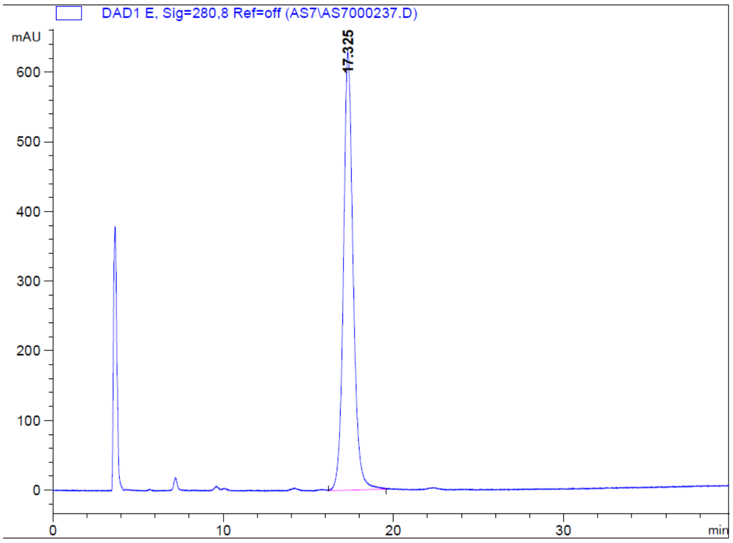

Peak #	RetTime [min]	Type	Width [min]	Area [mAU*s]	Height [mAU]	Area %
1	17.325	BB	0.5506	2.49048e4	628.14355	100.0000
Totals :				2.49048e4	628.14355	

Figure S19. HPLC of compound (*S*)-**9** ((S,S)-Whelk-O1, MeOH–H_2O 85 : 15, 0.8 mL/min; detection at 280 nm): *t* 17.3 min.

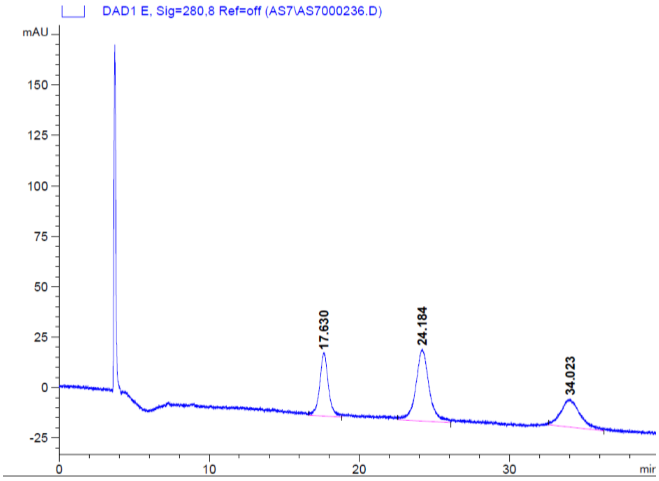

Peak #	RetTime [min]	Type	Width [min]	Area [mAU*s]	Height [mAU]	Area %
1	17.630	BV	0.4958	1267.48206	31.08143	27.1357
2	24.184	BB	0.7476	2180.08521	35.54097	46.6737
3	34.023	BV	1.0600	1223.34167	13.88373	26.1907
Totals :				4670.90894	80.50612	

Figure S20. HPLC of mixture of diastereomers **9** ((S,S)-Whelk-O1, MeOH–H_2O 85 : 15, 0.8 mL/min, detection at 280 nm): $t_{S,S}$ 17.6 min (27%), $t_{R*,S*}$ 24.2 min (47%), $t_{R,R}$ 34.0 min (26%).

Cytotoxicity Assessment

Table S1. Cell lines and composition of culture medium

Cell line	Composition of complete culture medium
**CT-26** murine colon carcinoma	RPMI-1640 supplemented with 100 × Glutamax, 100 × Penicillin-Streptomycin, 10% FBS, 0.1 M HEPES
**4T1** murine mammary carcinoma	DMEM/F12 (4.5 mg/L glucose, 25 mM HEPES), 100 × Glutamax, 100 × Penicillin-Streptomycin, 10% FBS
**MDA-MB-231** human breast adenocarcinoma	DMEM/F12 (4.5 mg/L glucose, 25 mM HEPES), 100 × Glutamax, 100 × Penicillin-Streptomycin, 10% FBS
**COLO201** human colorectal adenocarcinoma	RPMI-1640, 100 × Glutamax, 100 × Penicillin-Streptomycin, 10% FBS
**HepG2** human hepatocellular carcinoma	DMEM/F12 (4.5 mg/L glucose, 25 mM HEPES), 100 × Glutamax, 100 × Penicillin-Streptomycin, 10% FBS
**A549** human non-small-cell lung carcinoma	RPMI-1640 supplemented with 10% FBS, 2mM L-glutamine and 100 × Penicillin-Streptomycin
**SK-BR-3** human breast adenocarcinoma	RPMI-1640 supplemented with 10% FBS, 2mM L-glutamine and 100 × Penicillin-Streptomycin
**SNU-1** human gastric carcinoma	RPMI-1640 supplemented with 10% FBS, 2mM L-glutamine and 100 × Penicillin-Streptomycin
**Jurkat** human acute T-lymphoblastic leukemia	RPMI-1640 supplemented with 10% FBS, 2mM L-glutamine and 100 × Penicillin-Streptomycin
**WI-38** human lung fibroblasts	RPMI-1640 supplemented with 10% FBS, 2mM L-glutamine and 100 × Penicillin-Streptomycin

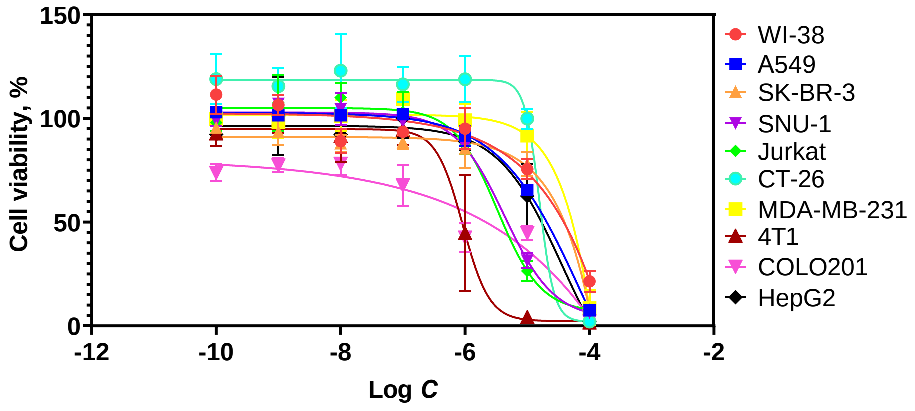

Figure S21. Viability of WI-38, A549, SK-BR-3, SNU-1, Jurkat, CT-26, MDA-MB-231, 4T1, COLO201, HepG2 cells *vs.* logarithmic concentration of compound **1c** after a 72-h incubation.

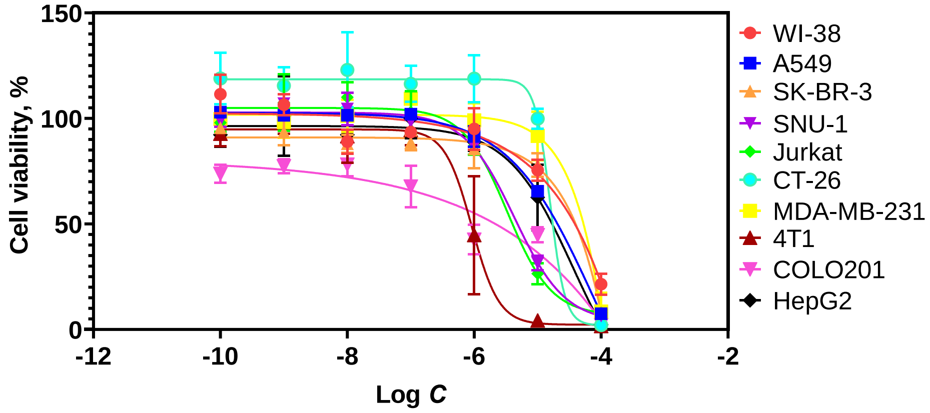

Figure S22. Viability of WI-38, A549, SK-BR-3, SNU-1, Jurkat, CT-26, MDA-MB-231, 4T1, COLO201, HepG2 cells *vs.* logarithmic concentration of compound **1d** after a 72-h incubation.

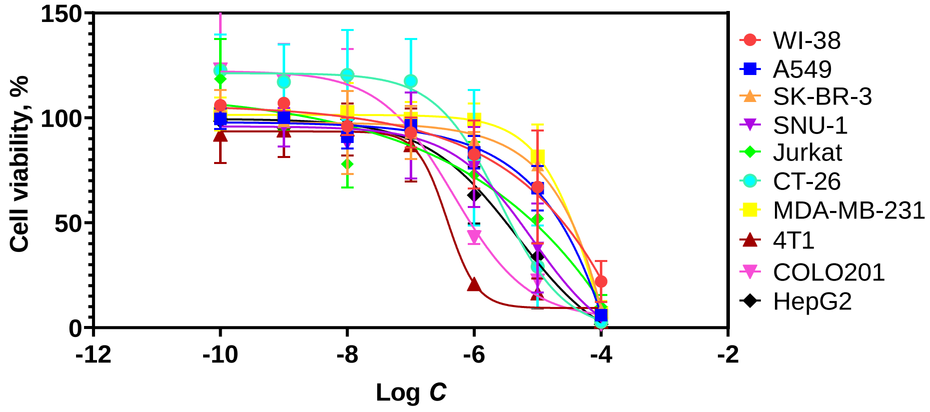

Figure S23. Viability of WI-38, A549, SK-BR-3, SNU-1, Jurkat, CT-26, MDA-MB-231, 4T1, COLO201, HepG2 cells *vs.* logarithmic concentration of compound **1e** after a 72-h incubation.

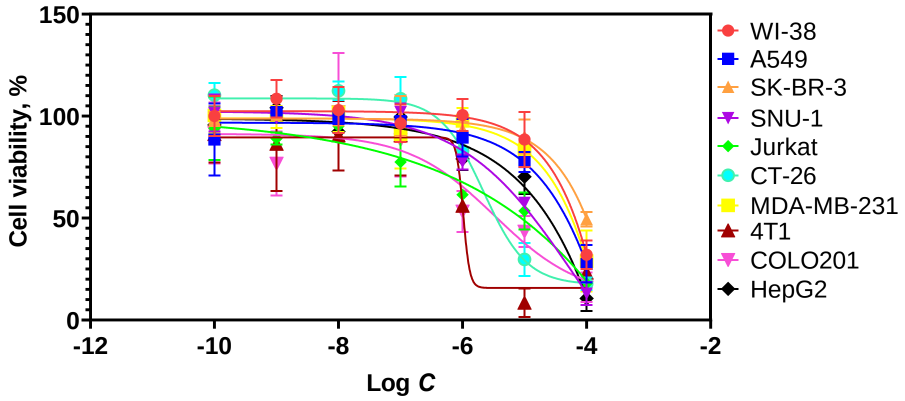

Figure S24. Viability of WI-38, A549, SK-BR-3, SNU-1, Jurkat, CT-26, MDA-MB-231, 4T1, COLO201, HepG2 cells *vs.* logarithmic concentration of compound **1f** after a 72-h incubation.

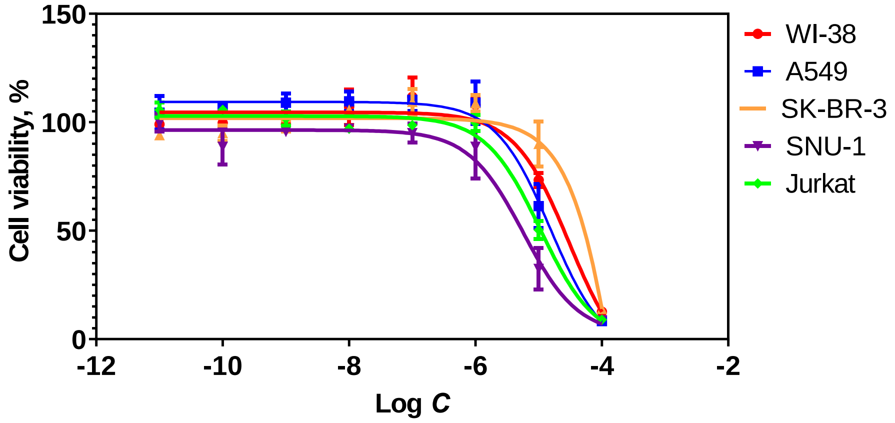

Figure S25. Viability of WI-38, A549, SK-BR-3, SNU-1, and Jurkat cells *vs.* logarithmic concentration of compound **6** after a 72-h incubation.

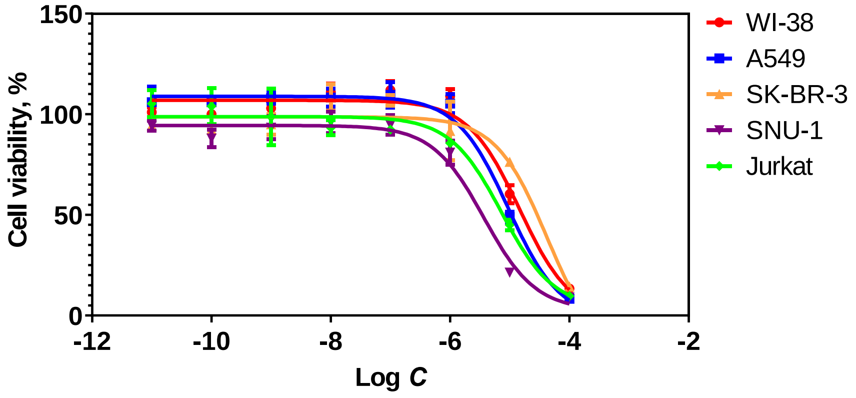

Figure S26. Viability of WI-38, A549, SK-BR-3, SNU-1, and Jurkat cells *vs.* logarithmic concentration of compound **7** after a 72-h incubation.

Cell Cycle Analysis

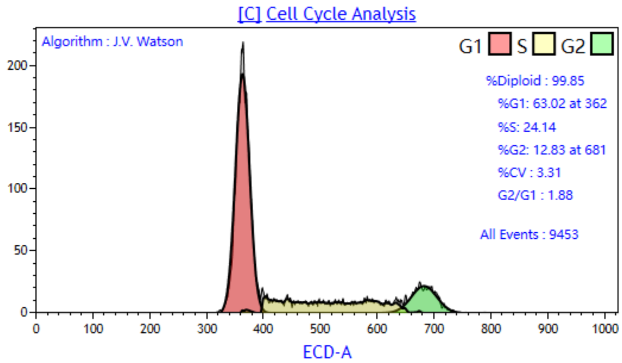

(a)

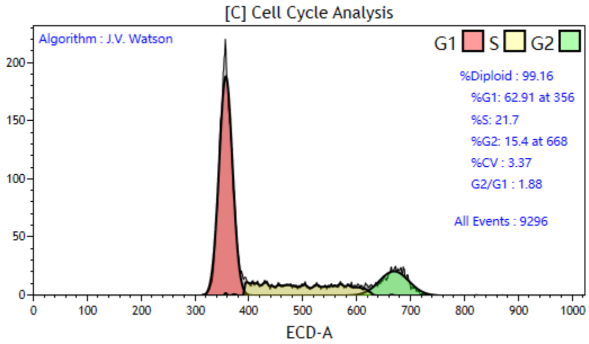

(b)

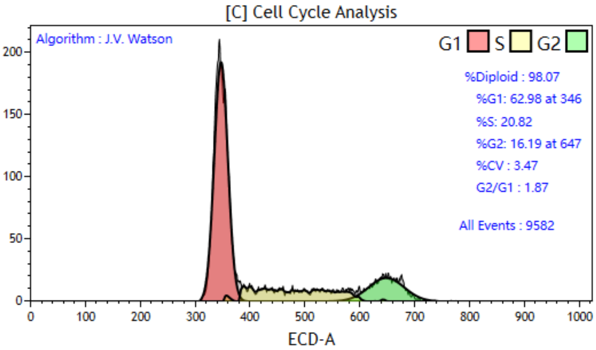

(c)

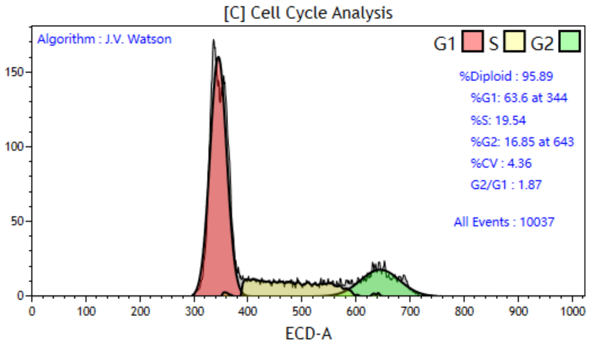

(d)

Figure S27. Diagrams of the COLO201 cell distribution by cell cycle phases: (**a**) without the addition of the test compound (control) and after 24-h incubation with compound **1d** at concentrations of (**b**) 0.9×10^{-6} M, (**c**) 1×10^{-6} M, and (**d**) 2×10^{-6} M.

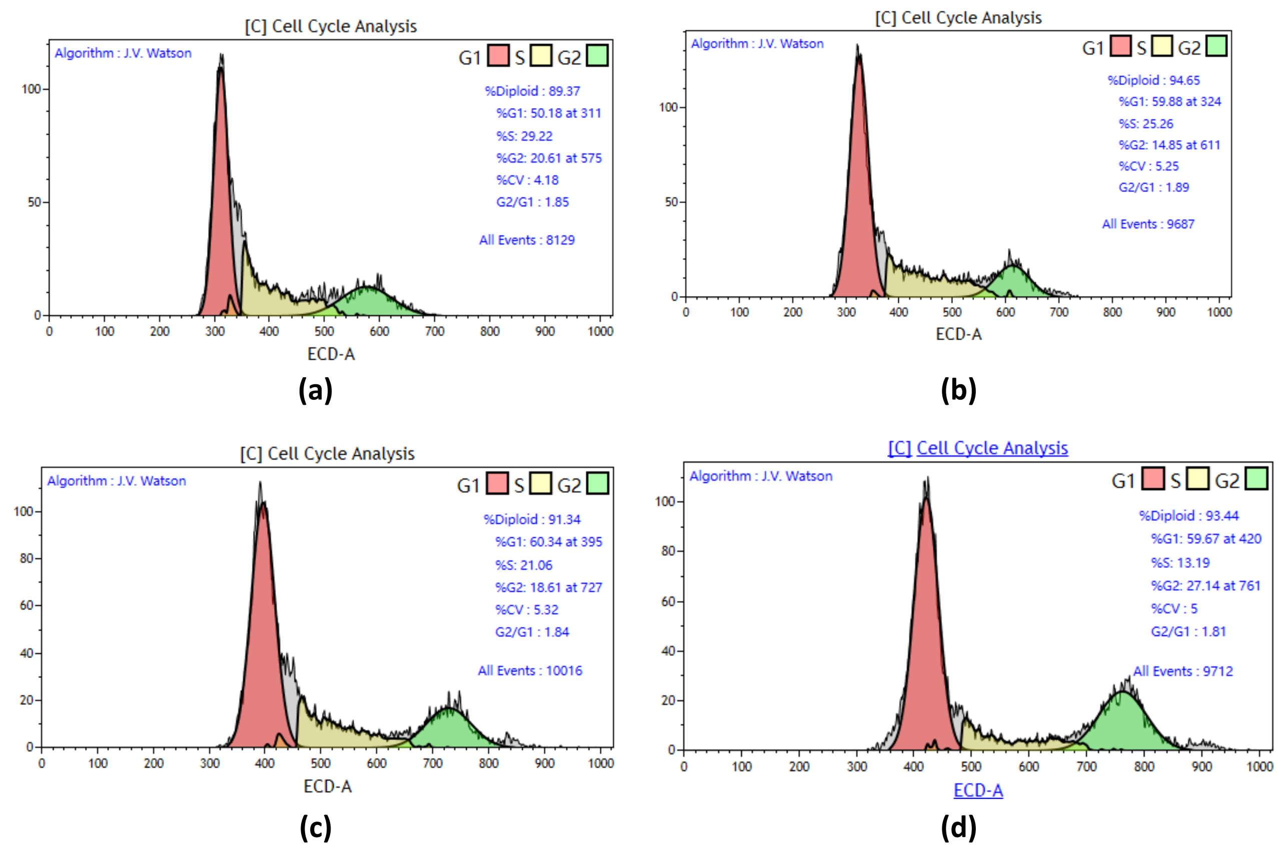

Figure S28. Diagrams of the MDA-MB-231 cell distribution by cell cycle phases: (**a**) without the addition of the test compound (control) and after 24-h incubation with compound **1d** at concentrations of (**b**) 10×10^{-6} M, (**c**) 20×10^{-6} M, and (**d**) 30×10^{-6} M.

Table S2. Effect of compound **1d** on the cell cycle of the COLO201 cells. Data are presented as $M \pm$ SD (n = 3).

Concentration (M)	Number of cells (%) in cell cycle phase			Cell proliferation index (%)
	G0/G1	S	G2M	
0	63.38±2.16	24.64±1.24	11.98±1.23	36.62±2.16
0.9×10^{-6}	62.95±0.21	21.35±0.34*	15.70±0.45*	37.05±0.21
1×10^{-6}	63.21±0.29	21.38±0.70*	15.41±0.74*	36.78±0.29
2×10^{-6}	64.60±0.97	18.51±0.99*	16.84±0.08*	35.35±0.96

* $p < 0.05$ compared to control.

Table S3. Effect of compound **1d** on the cell cycle of the MDA-MB-231 cells. Data are presented as $M \pm$ SD (n = 3).

Concentration (M)	Number of cells (%) in cell cycle phase			Cell proliferation index (%)
	G0/G1	S	G2M	
0	48.01±1.94	31.22±1.81	20.78±1.00	52.00±1.94
10×10^{-6}	60.92±0.91*	25.33±0.42*	13.74±1.13*	39.08±0.91*
20×10^{-6}	62.13±1.98*	20.52±0.81*	17.05±1.36*	37.57±1.98*
30×10^{-6}	59.37±1.02*	13.13±0.21*	27.49±1.22*	40.63±1.01*

* $p < 0.05$ compared to control.
